# Secretomes of apoptotic mononuclear cells ameliorate neurological damage in rats with focal ischemia

**DOI:** 10.12688/f1000research.4219.2

**Published:** 2014-10-28

**Authors:** Patrick Altmann, Michael Mildner, Thomas Haider, Denise Traxler, Lucian Beer, Robin Ristl, Bahar Golabi, Christian Gabriel, Fritz Leutmezer, Hendrik Jan Ankersmit

**Affiliations:** 1 Department of Thoracic Surgery, Medical University of Vienna, Vienna, 1090, Austria; 2 Christian Doppler Laboratory for Cardiac and Thoracic Diagnosis and Regeneration, Vienna, 1090, Austria; 3 Department of Dermatology, Medical University of Vienna, Vienna, 1090, Austria; 4 Section for Medical Statistics, Center for Medical Statistics, Informatics, and Intelligent Systems, Medical University of Vienna, Vienna, 1090, Austria; 5 Red Cross Transfusion Service for Upper Austria, Linz, 4017, Austria; 6 Department of Neurology, Medical University of Vienna, Vienna, 1090, Austria

## Abstract

The pursuit of targeting multiple pathways in the ischemic cascade of cerebral stroke is a promising treatment option. We examined the regenerative potential of conditioned medium derived from rat and human apoptotic mononuclear cells (MNC), rMNC ^apo sec^ and hMNC ^apo sec^, in experimental stroke.

We performed middle cerebral artery occlusion on Wistar rats and administered apoptotic MNC-secretomes intraperitoneally in two experimental settings. Ischemic lesion volumes were determined 48 hours after cerebral ischemia. Neurological evaluations were performed after 6, 24 and 48 hours. Immunoblots were conducted to analyze neuroprotective signal-transduction in human primary glia cells and neurons. Neuronal sprouting assays were performed and neurotrophic factors in both hMNC ^apo sec^ and rat plasma were quantified using ELISA.

Administration of rat as well as human apoptotic MNC-secretomes significantly reduced ischemic lesion volumes by 36% and 37%, respectively. Neurological examinations revealed improvement after stroke in both treatment groups. Co-incubation of human astrocytes, Schwann cells and neurons with hMNC ^apo sec^ resulted in activation of several signaling cascades associated with the regulation of cytoprotective gene products and enhanced neuronal sprouting *in vitro*. Analysis of neurotrophic factors in hMNC ^apo sec^ and rat plasma revealed high levels of brain derived neurotrophic factor (BDNF).

Our data indicate that apoptotic MNC-secretomes elicit neuroprotective effects on rats that have undergone ischemic stroke.

## Introduction

The search for clinically effective strategies to intercept the deleterious events that follow a stroke is ongoing. This quest has been particularly driven by the limitations that the use of tissue plasminogen activator (tPA) poses in patients with ischemic stroke ( Fonarow *et al.*, 2011). For Europe, it is projected that stroke events will increase from 20% in 2002 to 35% in 2050 in the population above 65 years of age ( Truelsen *et al.*, 2006). The United States report a yearly incidence of approximately 795,000, killing about 135,000 people each year ( Roger *et al.*, 2012). Most patients have to settle for the need of specialized care culminating into a burden to both persons affected and health care systems ( Strong *et al.*, 2007). Even though the potential of targeting neuroprotective pathways to treat ischemic stroke has been debated extensively, there seems to be a consensus towards more multilayered strategies ( Iadecola & Anrather, 2011).

Several neuroprotective proteins that play a role in the ischemic cascade have been identified and studied, such as cAMP response element-binding protein (CREB), Akt, extracellular-signal regulated kinase (Erk1/2), or heat shock protein 27 (HSP27) ( Qi *et al.*, 2012; Roux & Blenis, 2004). The transcription factor CREB, for instance, exerts its neuroprotective role in the ischemic response by activating protective genes and trophic factors such as B-cell lymphoma 2 (Bcl-2) or brain-derived neurotrophic factor (BDNF) ( Wilson *et al.*, 1996). The protein-chaperone HSP27 inhibits pathways leading to cell death ( van der Weerd *et al.*, 2010). Finding a treatment targeting these proteins simultaneously could open a new window of opportunity in acute stroke management and regeneration.

The role of different populations of adult stem cells is being investigated in several fields of regenerative medicine. Distant stem cells track sites of injury and counteract tissue damage ( Assmus *et al.*, 2011; Brunner *et al.*, 2008; Lagasse *et al.*, 2000). It is hypothesized that human mesenchymal stem cells (hMSCs) produce cytokines and growth factors that subsequently repair damaged tissues, including the brain ( Chen *et al.*, 2001). After homing to the injured areas, where hypoxia, apoptosis, and inflammation occur, hMSCs secrete trophic factors that enable endogenous repair ( Joyce *et al.*, 2010). Hence, the scientific community endeavored to either infuse or inject stem cells in multiple organ-specific disease entities with only limited clinical success in myocardial infarction ( Jensen & Patterson, 2013; Lemmens & Steinberg, 2013; Wollert & Drexler, 2010).

In 2005, *Thum et al.* postulated in their “dying stem cell hypothesis” that therapeutic stem cells are already in the state of apoptosis while being processed for treatment, thus causing immune suppression by scaling down the adaptive and innate immune system ( Thum *et al.*, 2005). The authors speculated that these “therapeutic apoptotic stem cells” are able to attenuate hypoxia-induced inflammation ( Fadok *et al.*, 2001; Saas *et al.*, 2010).

We have extended this concept and utilized suspensions of apoptotic peripheral blood mononuclear cells (MNCs), rather than stem cells themselves, as a therapeutic agent for the treatment of rodent myocardial infarction in a previous study ( Ankersmit *et al.*, 2009; Hoetzenecker *et al.*, 2012; Lichtenauer *et al.*, 2011). The almost complete absence of long term myocardial scarring led us to newly validate the suitability of peripheral MNCs and their secreted factors for regenerative medicine. In our previous work we showed that first, human apoptotic MNC-secretomes (hMNC ^apo sec^) circumvented inflammation and caused preferential homing of c-kit+/CD34- endothelial progenitor cells; second, hMNC ^apo sec^ caused immune suppression *in vitro* and, third, paracrine factors derived from human apoptotic MNC-secretomes led to an upregulation of matrix metalloproteinase 9 (MMP-9) and Interleukin 8 (IL-8) in primary cultured human fibroblasts. Both these factors are known to be involved in neoangiogenesis ( Nold-Petry *et al.*, 2010). In addition, hMNC ^apo sec^ have been shown to cause enhanced wound healing *in vivo* via the formation of new blood vessels and increased migration of primary cultured fibroblasts and keratinocytes ( Mildner *et al.*, 2013). Ultimately, one single intravenous administration of hMNC ^apo sec^ in a large animal closed chest reperfusion model of acute myocardial infarction (AMI) prevented myocardial damage ( Ankersmit *et al.*, 2009; Hoetzenecker *et al.*, 2012; Lichtenauer *et al.*, 2011).

With these data at hand, we investigated in the present study whether secretomes derived from rat (rMNC ^apo sec^) and human (hMNC ^apo sec^) apoptotic MNCs are also able to reduce ischemic lesion volumes and improve neurological outcome in a rat MCAO (middle cerebral artery occlusion) model.

## Materials and methods

### Ethics statement

All animal procedures were approved by the Animal Research Committee of the Medical University of Vienna (Protocol No.: 66.009/127-II/3b/2011) in accordance to the guidelines for the Care and Use of Laboratory Animals by the National Institutes of Health. Efforts were made to minimize suffering.

For the production of human MNC-secretomes, human MNCs were isolated from whole blood of healthy volunteers. This was approved by the ethics committee of the Medical University of Vienna (approval number: EK 2010/034). Participants provided their written informed consent.

### Animals

A total of 84 adult male Wistar Rats (Charles River Laboratories, Sulzfeld, Germany) weighing 280–320 g were used. Animals were kept in cages of three to four and accustomed to a 12 hour light-dark cycle for two weeks. Nutrition and tap water were provided ad libitum. Sixteen animals made up the secretome group for which MNCs were extracted out of whole blood and their secretomes produced. Ten animals were randomly selected for the pilot-phase with the intention of establishing protocol and attaining a consistent surgical technique. The remaining 58 animals were used for the study groups and randomly assigned to either the control or the treatment group. Deaths occurred equally in both study groups (total N=21, n=10 in setting 1 [rMNC ^apo sec^] and n=11 in setting 2 [hMNC ^apo sec^], 7 animals were excluded from the study (n=3 in setting 1 and n=4 in setting 2) and euthanized within 6 hours after MCAO due to severe dyspnea and suffering. For statistical analyses, the remaining 30 animals were used (n=16 in setting 1 and n=14 in setting 2) Data were analyzed in a blinded manner. Animals’ tails were marked with colored ink pens before surgery. From that moment on, investigators registered values using only that color code. Surgery was performed by a surgeon unaware if the animals received treatment or placebo. Neuroscore was evaluated by an investigator not involved in the surgical procedure or application of compounds. Statistical analyses were performed by an external statistician.

### Production of apoptotic MNC-secretomes from rats (rMNC ^apo sec^) and control medium for experimental setting 1

This section describes the production of apoptotic MNC-secretomes derived from rats ( Hoetzenecker *et al.*, 2013). These were used for the treatment group in setting 1 whereas the treatment group in setting 2 received apoptotic MNC-secretomes derived from humans (see below). Syngeneic rat-MNCs were harvested from splenocytes of Wistar rats. For this procedure, animals were anesthetized with an intraperitoneal administration of Ketamine (100 mg/kg) and Xylazine (10 mg/kg). Through a midline incision, spleens were harvested and MNCs were separated by passing spleens through 70 µm and 40 µm cell strainers (BD Biosciences, Vienna, Austria). Red blood cells were lyzed for 90 seconds using a red blood cell lysing buffer (Sigma Aldrich, Vienna, Austria). After washing, MNCs were resuspended in 4 mL CellGro serum-free medium (CellGenix GmbH, Freiburg, Germany) and apoptosis was induced by Caesium-137 irradiation (Department of Transfusion Medicine, Vienna General Hospital) with 45 Gy. Cells were cultivated in CellGro serum-free medium at a concentration of 25×10 ^6^ cells/mL at 37°C and 5% CO _2_ for 18 hours. Cells were removed by centrifugation at 1300 RPM for 9 minutes (Beckman Coulter Allegra ^®^ X-15R, Brea, CA, USA) and cell culture supernatants were then dialyzed against 50 mM ammonium acetate (Sigma Aldrich) using dialysis membranes (cut off: 6–8 kDa; Spectrum laboratories, Breda, The Netherlands) for 24 hours at 4°C on a shaking platter. Subsequently, the dialyzed supernatants were lyophilized over night (Lyophilizator Christ alpha 1–4, Martin Christ Gefriertrocknungsanlagen GmbH, Osterode am Harz, Germany). Lyophilization was performed at -20°C and 0.1 mbar pressure. The final product, rMNC ^apo sec^, was stored at -80°C. For the control group, the same cell culture medium that was used for the production of rMNC ^apo sec^ was irradiated, cultivated, dialyzed, and lyophilized accordingly. All cell and tissue samples were handled under sterile conditions. Microbial smears on chocolate agars (BD Biosciences, Vienna, Austria), a non-selective media for cultivation of fastidious microorganisms, were performed before lyophilization to rule out contaminations.

### Production of apoptotic MNC-secretomes from humans (hMNC ^apo sec^) and control medium according to GMP protocol for experimental setting 2

Pathogen-reduction methods such as photodynamic treatment with methylene blue (MB) plus visible light and gamma radiation have been developed to inactivate viruses and other pathogens in plasma and platelet concentrates. Regulatory authorities require these two pathogen reduction steps for blood derived products such as IVIg, plasma or coagulation factors ( Gauvin & Nims, 2010; Lambrecht *et al.*, 1991; Nims *et al.*, 2011; Wallis & Melnick, 1965) to be performed in a good manufacturing practice (GMP) facility.

Human apoptotic MNC-secretomes were prepared as described previously ( Lichtenauer *et al.*, 2011). Briefly, venous blood samples (75 mL) were drawn from healthy volunteers (n=15). Blood cells were separated using Ficoll-Paque (GE Healthcare Bio-Sciences AB, Stockholm, Sweden) density gradient centrifugation. MNCs were resuspended with CellGro serum-free medium. After irradiation with 45 Gy as described above, cells were cultivated with CellGro serum-free medium at a concentration of 25×10 ^6^ cells/mL under sterile conditions for 18 hours. Supernatants were collected by centrifugation and methylene blue (MB) plus light treatment was performed in the Theraflex MB-Plasma system (MacoPharma, Mouvaux, France) using the Theraflex MB-Plasma bag system (REF SDV 0001XQ) and an LED-based illumination device (MacoTronic B2, MacoPharma). Light energy was monitored and reached 120 J/cm ^2^. Integrated in the bag system was a pill containing 85 mg of MB, yielding a concentration range of 0.8 to 1.2 mM MB per unit. Removal of MB and photoproducts by Blueflex filtration was done consecutively after treatment. Air was removed from the plasma units before illumination. After lyophilization of this viral inactivated cell culture supernatant as indicated above, the lyophilized powder ran through the second step of pathogen removal, gamma irradiation. Gamma irradiation was performed by Mediscan, which operates a gamma irradiation unit (Gammatron 1500, Mediscan, Seibersdorf, Austria). Gamma rays were generated by radioactive decay of Cobalt 60. For this purpose, apoptotic MNC-secretomes were put in metal sterilization totes that pass on a meandering path through the irradiation vault around the emitting center in five layers. The cobalt unit emits photons that are almost isotropic. Regarding the complex path (280 positions in five layers) the dose is distributed consistently. The dose rate recorded by a Polymethyl methacrylate (PMMA) dosimeter was determined to be 25000 Gy after 23 hours of irradiation. The lyophilized and two-step pathogen-free supernatant of apoptotic MNCs (hMNC ^apo sec^) was stored at -80°C. For the control group, cell culture medium was put through the same steps (cultivation, two step pathogen reduction, irradiation and lyophilization).

### Verification of apoptosis in cultured irradiated apoptotic rMNCs using flow cytometry

Syngeneic rMNCs (MNCs derived from rats) were harvested from splenocytes of Wistar rats (n=5) using the same protocol as described earlier. To investigate apoptosis rates during the production of our compounds, washed MNCs that had been cultured for 18 hours were divided into two groups, one to be irradiated in order to induce apoptosis (as described before by using Caesium-137 irradiation with 45 Gy) the other to remain non-irradiated. Except for irradiation, MNCs in both groups were processed equivalently (cultivation, dialysis, lyophilization). Co-staining with Annexin-V/Propidium Iodide (FITC/PI, Becton Dickinson, Franklin Lakes, NJ, USA) was performed following manufacturer’s instructions. The rate of apoptosis was then evaluated on a flow cytometer (FC500, Coulter, CA, USA).

### Animal preparation and induction of focal cerebral ischemia

Fifty eight animals were weighed and anesthetized with an intraperitoneal administration of Ketamine (100 mg/kg) and Xylazine (10 mg/kg). This was followed by a subcutaneous injection of Piritramide (15 mg/kg). Animals were then intubated with an 18G intravenous catheter (BD Biosciences) and anesthesia was maintained throughout surgery with 1.5% isoflurane delivered in 1.5 L air and 0.8 L oxygen per minute. Body temperature was regulated using a heating pad (Trixie 76085 Heizmatte, Trixie, Flensburg, Germany). Permanent middle cerebral artery occlusion (MCAO) of the right hemisphere was performed according to the suture model described by Zea Longa *et al.* using a coated monofilament (Doccol Corporation, CA, USA) ( Longa *et al.*, 1989). Briefly, a 3 cm coated monofilament with a thickened tip was inserted into the external carotid artery (ECA) and advanced to the middle cerebral artery (MCA) to induce ischemia in the MCA territory.

### Postoperative administration of apoptotic MNC-secretomes

**Figure 1.**
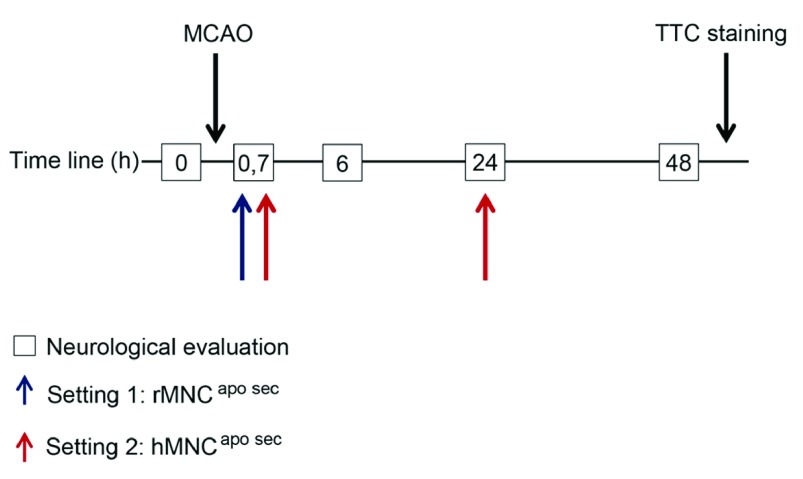
Experimental study setting. For setting 1, rMNC ^apo sec^ (apoptotic MNC-secretomes from rats) were injected 40 minutes after MCAO (blue arrow). In setting 2, hMNC ^apo sec^ (apoptotic MNC-secretomes from humans) were administered twice at 40 minutes (0.7 hours) and 24 hours after MCAO (red arrows). In both settings, neurological evaluations were performed at 0 hours (before surgery) as well as 6, 24, and 48 hours after surgery (boxes). Both treatment and control animals were euthanized 48 hours after surgery and brain slices were treated with TTC (2,3,5-triphenyltetrazolium chloride) to stain ischemic areas in the brain.

In the first experimental setting, lyophilized rMNC ^apo sec^ (produced from 12.5×10 ^6^ apoptotic rat MNCs) or control medium was resuspended in 0.3 mL saline (Fresenius Kabi, Vienna, Austria) in the laboratory prior to surgery. In order to investigate the potency of apoptotic MNC-secretomes, both the treatment group and the control group (n=29) randomly received 0.3 mL rMNC ^apo sec^ or control medium intraperitoneally forty minutes after surgery ( Figure 1, blue arrow). In the second setting, lyophilized hMNC ^apo sec^ (produced from 12.5×10 ^6^ apoptotic human MNCs) or lyophilized control medium were each resuspended in the laboratory in 0.3 mL saline prior to surgery. In order to investigate whether a higher dosage and time interval would provide additional benefits, animals from experimental setting 2 (n=29) received two intraperitoneal doses 40 minutes and 24 hours after MCAO induction ( Figure 1, red arrows). The rationale for this two-step-approach is given in the discussion. In addition, all animals were given a subcutaneous injection of 3.5 mL/kg saline after surgery and put under a heating lamp until they woke up.

### Measurement of infarct volumes

**Figure 2.**
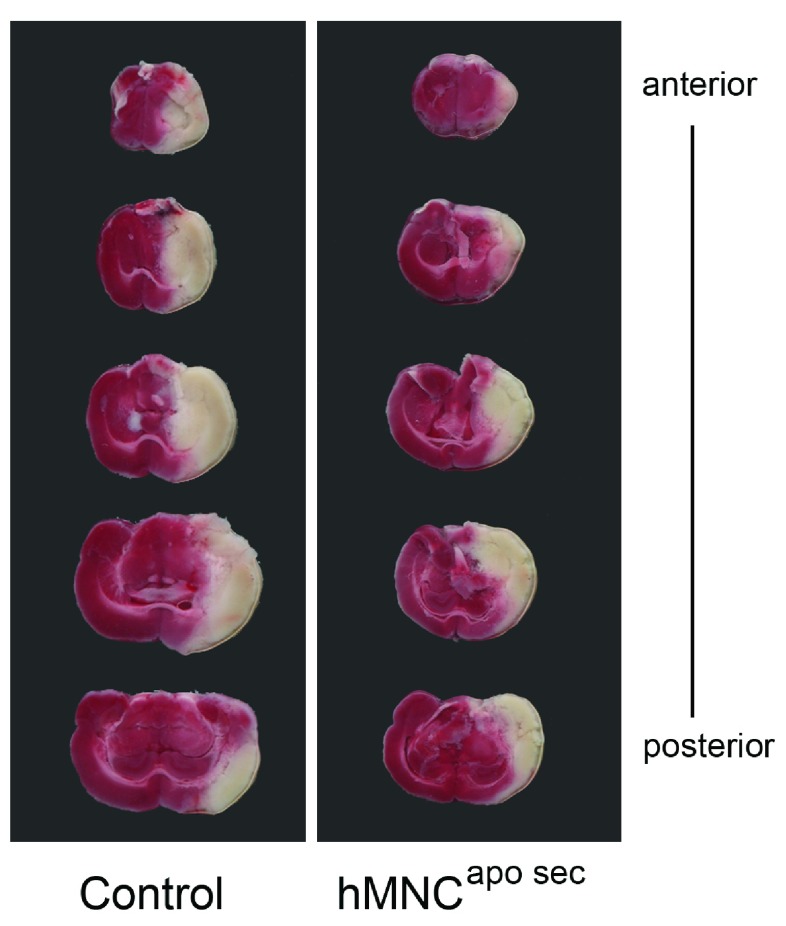
Representative brain slices of rats subjected to MCAO. Brains were stained with a 2% solution of TTC forty-eight hours after MCAO. Animals received either treatment (in this representative scan: hMNC ^apo sec^) or control medium, in this case, 40 minutes 24 hours after surgery. White areas indicate ischemic tissue while red areas stain for non-ischemic tissue. Animals treated with control medium (left image) had larger ischemic (=white) areas than animals treated with hMNC ^apo sec^ (right image).

In both experimental settings, animals were euthanized 48 hours after surgery with an intraperitoneal injection of 600 mg/kg Pentobarbital. Brains were harvested and cut into five 2 mm coronal slices using a brain matrix (Zivic Instruments, Pittsburgh, PA, USA) and razor blades (Zivic Instruments). In order to stain ischemic areas, brain slices were then incubated for 30 minutes at 37°C in a 2% solution of 2,3,5-triphenyltetrazolium chloride (TTC; CarlRoth, Karlsruhe, Germany) ( Bederson *et al.*, 1986). Slices were digitalized using a commercially available photo scanner (Epson Perfection V330 Scanner; Figure 2). Lesion volumes were determined by a blinded investigator using ImageJ planimetry software (Version 1.6.0_10; Rasband, W.S., ImageJ, U.S. National Institutes of Health; Bethesda, MD, USA). Lesion volumes were calculated with respect to edema formation using the following formula: 100×(Volume of the contralateral hemisphere-Volume of the ipsilateral hemisphere)/(Volume of the contralateral hemisphere). Ipsi- and contralateral lesion volumes were calculated by multiplication of area with slice thickness summed for all sections ( Swanson *et al.*, 1990).

### Neurological evaluation

Neurological examinations were performed by a blinded investigator in both experimental settings using a neurological score before surgery, and 6, 24, and 48 hours after surgery. The test at each time point consists of seven exercises and animals would receive a score ranging from 0 points (no pathological responses) to 7 points (maximum impairment). A successfully completed exercise would add 0 points to the score. Pathological performance in an exercise would add 1 point. The exercises were: left forepaw extension, instability to lateral push from right, tail hanging, walking on ground, whisker movement on the left, hearing, and vision ( Nedelmann *et al.*, 2007). E.g. if the rats could not hold against a hand pushing them from the right, they would get 1 point. If they did push against the examiner’s hand, they would get 0 points. Accordingly, if the rats did extend their left forepaw when hauled up by their tail, they would get 0 points. If they could not extend their left forepaw, they would get 1 point. This adds up to a score ranging from 0 points (=no pathological response at all), to 7 points (=highly impaired animal).

### Cell culture

Human primary astrocytes, Schwann cells and neurons were obtained from CellSystems (CellSystems Biotechnologie, St. Katharinen, Germany) and cultured in their respective growth medium (CellSystems) at 37°C and 5% CO _2_.

### Western blot analysis

3×10 ^6^ astrocytes, Schwann cells and 3×10 ^5^ neurons were seeded in 6-well plates (Costar, Vienna, Austria) and cultured overnight in their respective growth medium. After removal of the medium, cells were washed twice at room temperature with PBS (Gibco BRL, Gaithersburg, MA, USA) and cultured in their respective basal medium (Astrocyte or Schwann cell growth medium (CellSystems) without growth supplements) for 3 hours. Aliquots of lyophilized human MNC-secretome and control medium were resolved in the different basal media at a 10-fold concentration (lyophilized secretome, derived from 25×10 ^6^ cells/mL). One tenth of this solution was then directly added to the cell cultures. After 1 hour, the cells were washed at room temperature with PBS and lyzed in 200 µL SDS-PAGE loading buffer (100 mL contain: 1g SDS (Sigma, Vienna, Austria), 3 mg EDTA (Sigma) and 0.75g TRIS (Sigma). pH is adjusted with HCl (Merck, Vienna, Austria) to 6.8 for 10 minutes at room temperature. After sonication (Laborpartner, Vienna, Austria: output = 100%; 20 Cycle for 1 second each) and centrifugation (20000g for 10 minutes) proteins were size-fractionated by SDS-PAGE through an 8 to 18% gradient gel (Amersham Pharmacia Biotech, Uppsala, Sweden) and transferred to nitrocellulose membranes (BioRad, Hercules, CA, USA). Immunodetection was performed with anti-c-Jun (Cell Signaling Technology, Inc. Danvers, MA, USA; 1µg/mL, #9165), anti-phospho-c-Jun (Cell Signaling Technology; 1µg/mL, #9261), anti-CREB (Cell Signaling Technology; 1µg/mL, #9197), anti-phospho-CREB (Cell Signaling Technology; 1µg/mL, #9198), anti-Akt (Cell Signaling Technology; 1µg/mL, #2938), anti-phospho-AKT (Cell Signaling Technology; 1µg/mL, #9271), anti-Erk1/2 (Cell Signaling Technology; 1µg/mL, #4695), anti-phospho-Erk1/2 (Cell Signaling Technology; 1µg/mL, #4376), anti-HSP27 (Cell Signaling Technology; 1µg/mL, #2402), anti-phospho-Hsp27 (Cell Signaling Technology; 1µg/mL, #2404) followed by an HRP-conjugated goat anti-mouse IgG antiserum or a goat anti-rabbit IgG antiserum (GE Healthcare, Freiburg, Germany). Reaction products were detected by chemiluminescence with the ChemiGlow reagent (Biozyme Laboratories Limited, South Wales, U.K.) according to the manufacturer’s instructions.

### Neuronal sprouting assay

To investigate neuronal sprouting of human primary neurons, 1×10 ^4^ cells (CellSystems) were seeded in 24-well plates (Costar) and allowed to adhere for 24 hours. Cells were further cultivated in neuronal medium (see above) without growth factors for five days together with the secretome of hMNC derived from 2.5×10 ^6^ cells/mL (hMNC ^apo sec^) or control medium. After five days cells were fixed at room temperature in 100% methanol for 10 minutes and stained with methylene blue (Sigma; 0.5% in methanol). Excess methylene blue was washed out with distilled water, and culture wells were evaluated with an inverted microscope (EvosXL, Life Technologies, Carlsbad, CA, USA). Cell cultures were digitalized using an Olympus Digital Camera E-520 (3648×2736 pixels). A blinded observer set a random representative area of the photographed cell culture (as seen in Figure 7b) using Adobe Photoshop Lightroom Software (Version 5.2, 2013; Adobe, San José, CA, USA). (i) on a photograph of cells treated with human apoptotic MNC-secretomes and (ii) on a photograph showing cells treated with control medium. The areas in each photograph measured 1420×2456 pixels. Subsequently, they picked and marked visible distinct, full-length and non-overlapping 30–35 neurites using ImageJ software (Bethesda, MD). Another blinded investigator measured these marked neurons using ImageJ software.

### Determination of neurotrophic factors in apoptotic MNC-secretomes and control medium

BDNF, nerve growth factor (NGF) and glial derived neurotrophic factor (GDNF) in rMNC ^apo sec^, hMNC ^apo sec^, and control medium, were measured using commercially available ELISA-kits (Enzyme linked immunosorbent assay; BDNF: catalog# DY248; beta-NGF catalog# DY256; GDNF catalog# DY212; R&D Systems, Minneapolis, MN, USA). All samples were assayed in triplicates. Manufacturer’s instructions were followed and plates were read at 450 nm on a Wallac Multilabel counter 1420 (PerkinElmer, Boston, MA, USA).

### Determination of BDNF in rat plasma

To see whether intraperitoneal administration of apoptotic MNC-secretomes and control medium influence BDNF production, six rats were injected intraperitoneally with hMNC ^apo sec^ (n=3), or control medium, (n=3). Rats were euthanized with an intraperitoneal injection of 600 mg/kg Pentobarbital 24 hours after injection with hMNC ^apo sec^ (secretomes of 12.5×10 ^6^ cells) or control medium. Blood was retrieved in heparinized tubes, centrifuged, and plasma was stored at -20°C. Rat BDNF levels were measured using a commercially available BDNF-ELISA (catalog# KA0330, Abnova, Taipei, Taiwan) and plates were read at 450 nm on a Wallac Multilabel counter 1420 (PerkinElmer, Boston, MA, USA).

### Statistical analyses

To test for differences in lesion volume between control and treatment groups the Mann-Whitney *U-*test was applied. Differences were also assessed graphically using box-plots. The calculations were performed separately for each experimental setting. Linear mixed models were calculated to explain neuroscores by time point, treatment and interaction effects between time point and treatment. A random intercept term was included for each individual animal to account for the correlation of observations within an individual. The calculations were done using the MIXED procedure in SAS 9.3. The neuroscores were also assessed graphically by plotting a time curve of mean neuroscore values±SD for each group. Neurite lengths in neuronal cultures were compared applying the student’s t-test. A *p*-value of 0.05 or below was considered significant.

## Results

### Apoptosis rates in irradiated apoptotic rMNCs

**Figure 3.**
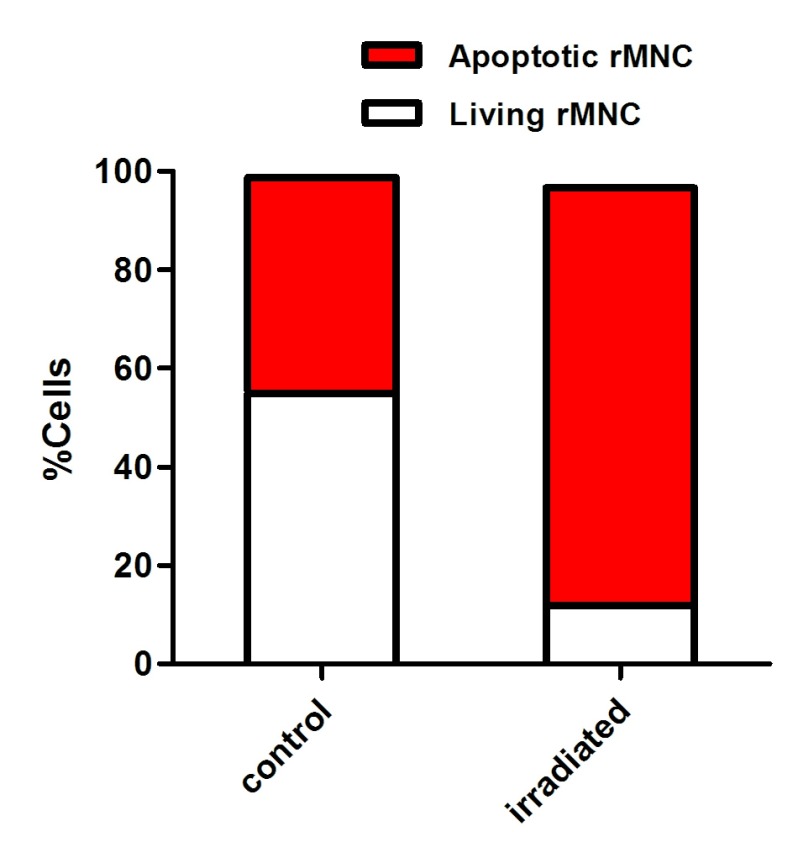
Apoptosis rates in irradiated and non-irradiated cultured rMNCs. Mean percentage values of viable (white) and apoptotic (red) rMNCs are given in this bar graph. The percentage of cells in either state are shown for non-irradiated rMNCs (control) and irradiated rMNCs after 18 hours in cell culture. Irradiated rMNCs correspond to the compound we used throughout the study.

In order to see the extent of apoptosis in rMNCs that had been cultured for 18 hours, we analyzed apoptosis rates in cultured irradiated and cultured non-irradiated rMNCs from 5 donors using flow cytometry. Analysis of cultured irradiated rMNCs revealed apoptosis rates of 85±5% (mean±SD) while 12±5 did not stain for FITC/PI, thus being viable. In the non-irradiated rMNC control group, 44±7 cells were apoptotic with 55±7 still being viable ( Figure 3).

### Apoptotic MNC-secretomes reduce the infarction volume in an experimental MCAO model

**Figure 4.**
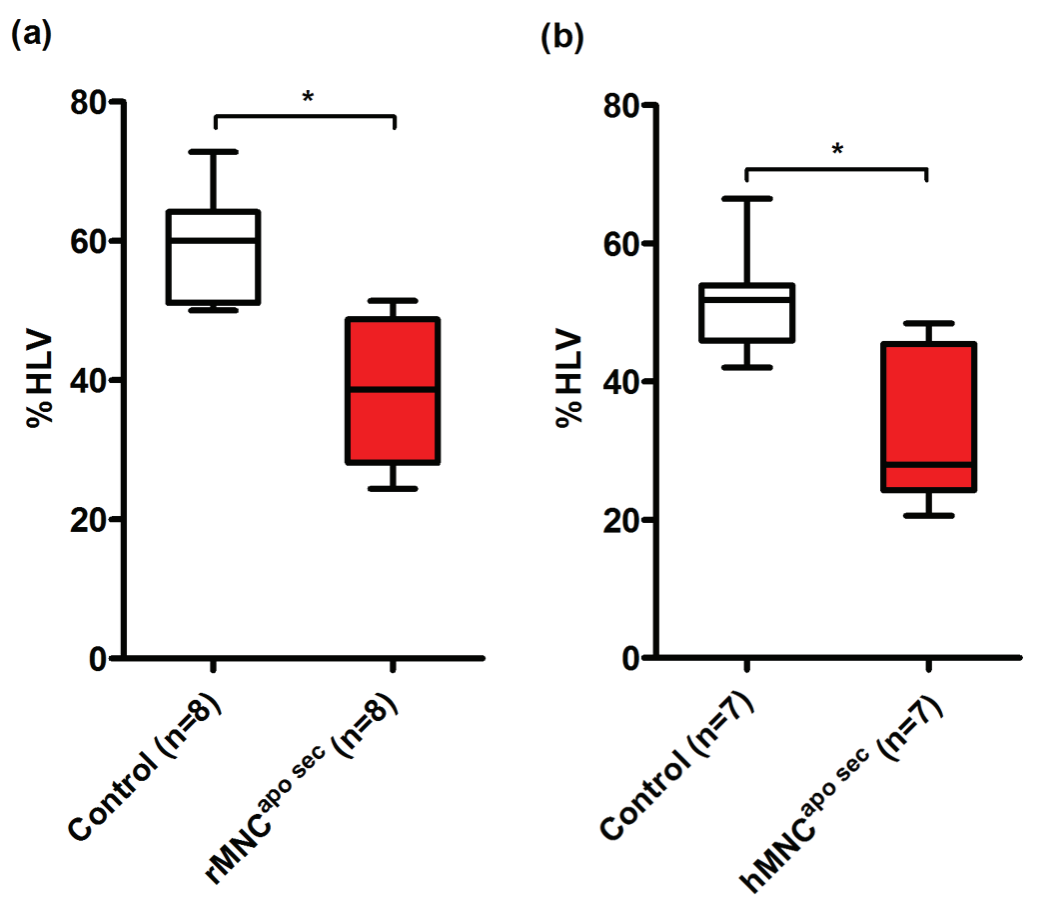
Infarct volumes in control animals and animals treated with apoptotic MNC-secretomes. The percentage of hemispheric lesion volumes (%HLV) are represented as box and whiskers plots, wherein the boxes indicate the 1 ^st^ and the 2 ^nd^ quartile and the whiskers the minimum and maximum within 1.5 times the interquartile range from the box. Figure 4a shows lesion volumes as the extend of ischemia in setting 1, where apoptotic MNC-secretomes derived from rats were administered 40 minutes after MCAO compared to controls. Figure 4b corresponds to setting 2, where apoptotic MNC-secretomes derived from humans were administered 40 minutes and 24 hours after MCAO. In both settings, MNC ^apo sec^ (red boxes) caused a significant decrease in infarct volumes (* *p*=0.0006 for setting 1, Figure 4a, and * *p*=0.0041 for setting 2, Figure 4b) compared to the control group (white boxes) that received only cell culture medium.

In a rat model of MCAO we examined the potential of apoptotic MNC-secretomes to reduce ischemic lesion volumes in an allogeneic (experimental setting 1, rMNC ^apo sec^) and a xenogeneic approach (experimental setting 2, hMNC ^apo sec^) ( Figure 1). The results of the allogeneic setting displayed significantly lower lesion volumes in the treatment group compared to the control group as shown by TTC-staining ( Figure 4). Treatment with rMNC ^apo sec^ led to a mean decrease of 36% in total infarct volume. Hemispheric lesion volumes (mean±SD) in the control group were 59%±8% ranging from 50% to 73% (Mann Whitney *U*-test; * *p*=0.0006; Figure 4a). The treatment group had a mean hemispheric lesion volume of 38%±11% ranging from 24% to 51%. In the xenogeneic setting, we injected hMNC ^apo sec^ 40 minutes and 24 hours after MCAO. The reduction of the infarction volume in the xenogeneic setting was statistically significant and comparable to that observed in the allogeneic setting (Mann Whitney *U*-test; * *p*=0.0041; Figure 4b). The mean decrease in total infarct volume was 37%. Hemispheric lesion volumes (mean±SD) in the control group were 52%±8% ranging from 42% to 67%. The treatment group had a mean hemispheric lesion volume of 33%±11% ranging from 21% to 48%.

### Apoptotic MNC-secretomes improve neurological outcome in an experimental MCAO model

In order to discover the effects of apoptotic MNC-secretomes on the neurological outcome, we performed a neurological exam on each animal at 4 specific time points. The first score was measured prior to surgery (baseline; 0 hours) and was 0 points for all animals. This was followed by 3 postoperative measurements at 6, 24 and 48 hours. All animals expressed neurological stroke-symptoms immediately after anesthesia wore off. The fixed effect coefficient estimates of the mixed models, their standard errors and *p*-values are shown in Table 1a and Table 1b. The factor Treatment was coded in a way that Treatment=0 corresponds to the control group and Treatment=1 corresponds to the treatment group. The time point 6 h is the reference group for the factor Time.

**Table 1.** Fixed effect coefficient estimates of the mixed models and their standard errors. Data include both experimental setting 1 that used apoptotic MNC-secretomes derived from rats, “rMNC ^apo sec^”, ( Table 1a), and setting 2 that used apoptotic MNC-secretomes derived from humans, “hMNC ^apo sec^”, ( Table 1b). *p*-values are calculated from t-tests with the null hypothesis of the true coefficient being equal to 0. The factor Treatment was coded in a way that Treatment=0 corresponds to the control group and Treatment=1 corresponds to the treatment group. The time point 6 h is the reference group for the factor Time.

Table 1a: Linear mixed model analysis for setting 1. “rMNC ^apo sec^”.			
Effect	Estimate	Standard Error	p
Intercept	5	0.2502	<.0001
Time 24 h	0	0.2566	1
Time 48 h	-0.1875	0.2566	0.471
Treatment	-0.0625	0.3538	0.8611
Treatment*Time 24 h	-1.0625	0.3629	0.0067
Treatment*Time 48 h	-1.5625	0.3629	0.0002
Table 1b: Linear mixed model analysis for setting 2. “hMNC ^apo sec^”.			
Effect	Estimate	Standard Error	p
Intercept	4.5714	0.2761	<.0001
Time 24 h	0.1429	0.3442	0.6818
Time 48 h	-0.5	0.3442	0.1593
Treatment	0	0.3905	1
Treatment*Time 24 h	-1.4286	0.4868	0.0072
Treatment*Time 48 h	-1.6429	0.4868	0.0025

**Figure 5.**
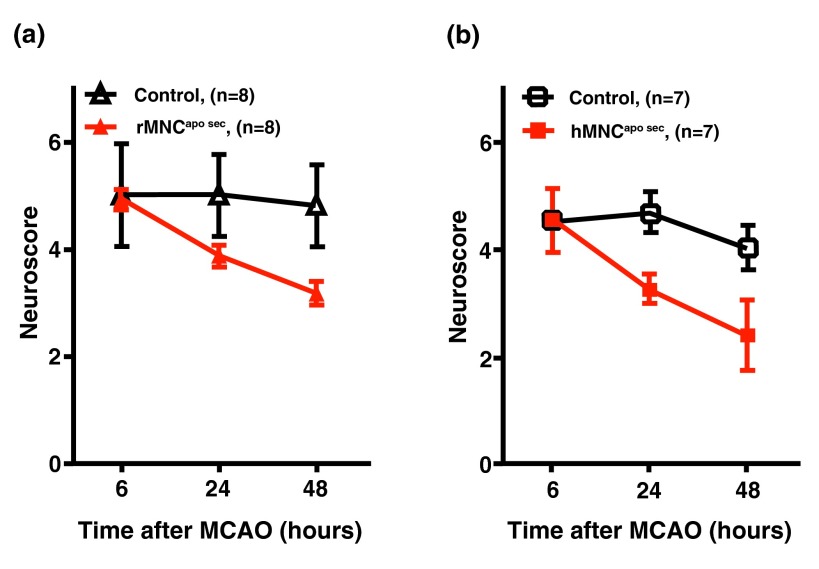
Neurological outcome score in control animals and animals treated with apoptotic MNC-secretomes. Mean neuroscores (±SD) are plotted over time. Treated animals (red triangles for setting 1, Figure 5a, and red squares for setting 2, Figure 5b) improved over time compared to controls (black/white triangles for setting 1, Figure 5a, and black/white squares for setting 2, Figure 5b). Error bars correspond to +/- one standard deviation.

The results from both settings are similar. At time point 6 h there is no significant mean difference in the neuroscores between control and treatment, which is suggested by the coefficient for Treatment. The coefficients for Time 24 h and Time 48 h are not significantly different from zero. We can therefore not conclude that the mean neuroscore in the control group changes with time. The interaction terms for Treatment and Time, however, are significant. This indicates a significant decrease in the mean neuroscore over time in the treatment group. These results correspond well to the graphical depictions of the time curves ( Figures 5a and 5b).

### Apoptotic MNC-secretomes activate signaling cascades involved in cytoprotection in glia cells

**Figure 6.**
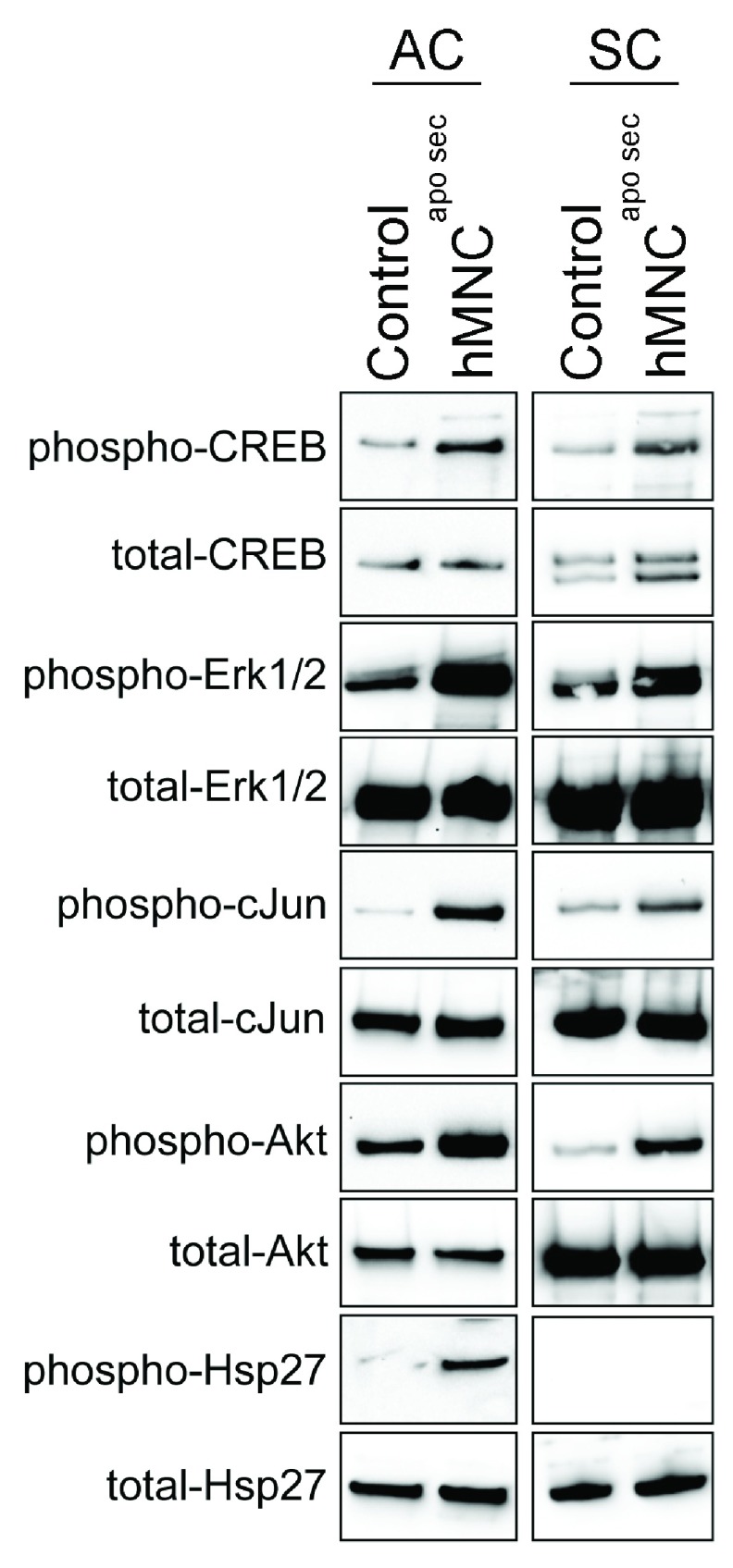
Expression of cytoprotective proteins in human Astrocytes **(AC)** and Schwann Cells (SCs). Cell extracts were prepared after stimulation with hMNC ^apo sec^ or cell culture Medium as control. Western blot analysis revealed an activation of CREB, ERK1/2, c-Jun, Akt, and HSP27. Proteins were normalized to the respective non-phosphorylated proteins.

Glia cells are non-neuronal cells that provide support and protection for neurons in the brain and peripheral nervous system ( Giaume *et al.*, 2010). We therefore investigated the potency of hMNC ^apo sec^ to activate/phosphorylate signaling-molecules that are part of protective pathways in human primary astrocytes as well as in Schwann cells. For Western blot analysis, astrocytes and Schwann cells were treated with hMNC ^apo sec^ for 1 hour. Both cell types showed an increased phosphorylation of CREB, Erk1/2, c-Jun, and Akt. Phosphorylation of HSP27 was only detected in astrocytes ( Figure 6).

### Apoptotic MNC-secretomes induce CREB phosphorylation and neuronal sprouting in human primary neurons and contain BDNF

**Figure 7.**
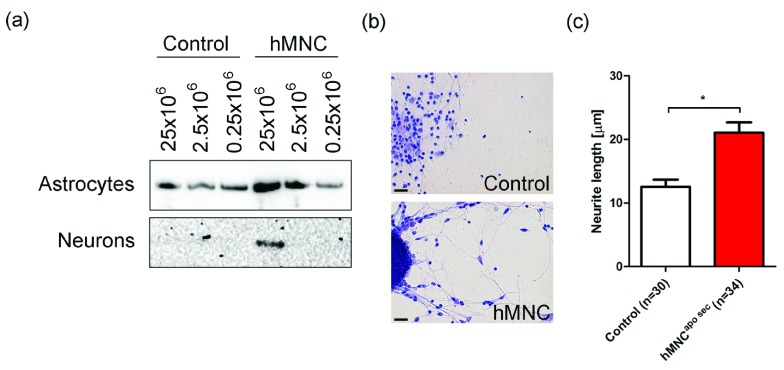
Enhanced CREB phosphorylation and neurite length in neurons treated with apoptotic MNC-secretomes. ( **a**) Cell extracts were prepared after stimulation with hMNC ^apo sec^ or cell culture medium as control. Western blot analysis for phospho-CREB revealed a dose dependent activation of CREB in astrocytes and in neurons. ( **b**) Neuron cultures treated with hMNC ^apo sec^ or control medium for five days were stained with methylene-blue. One representative picture of ten is shown. Bar=10 µm ( **c**) Lengths of neurons treated with hMNC ^apo sec^ or cell culture medium as control were calculated using ImageJ software. Bars represent the mean of five different cultures.

We next investigated whether hMNC ^apo sec^ are also effective in human primary neuron cultures. Western blot analysis of neurons and astrocytes revealed a rapid dose dependent activation of CREB phosphorylation ( Figure 7a). Incubation of neurons with apoptotic MNC-secretomes led to a significant increase in the length of newly sprouting neurons. Cultured neurons treated with hMNC ^apo sec^ had a mean neurite length of 21±1µm (mean±SEM) versus 13±1µm in neurons treated with control medium (t-test; * *p*<0.0001; Figure 7b–c).

**Figure 8.**
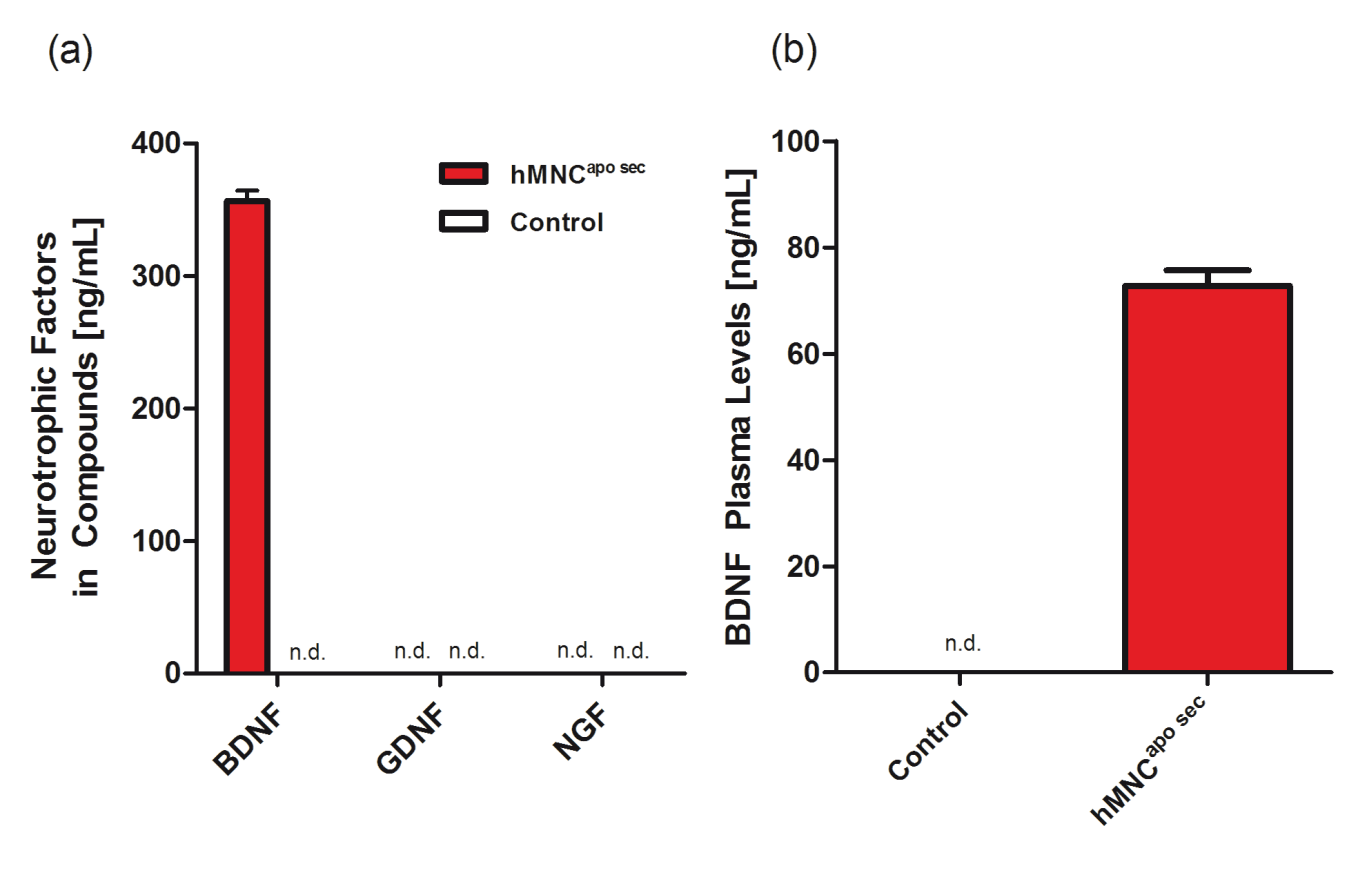
Profile of neurotrophic factors in hMNC ^apo sec^ and animals treated with hMNC ^apo sec^. ( **a**) ELISA for BDNF, GDNF, and NGF detected only levels of BDNF (356±14pg/mL, mean±SEM) in hMNC ^apo sec^. ( **b**) Six animals received an intraperitoneal injection with hMNC ^apo sec^ (n=3, red bar) or control medium (n=3, black/white bar) and BDNF plasma levels were determined 24 hours after administration using ELISA.

In order to characterize the composition of neurotrophic factors present in hMNC ^apo sec^, we performed ELISA for BDNF, GDNF and NGF. Interestingly, only high amounts of BDNF (356±14pg/mL) were detected in hMNC ^apo sec^, suggesting an exclusive role for this neurotrophic factor in hMNC ^apo sec^ ( Figure 8a).

### Effects of apoptotic MNC-secretomes on plasma BDNF-levels in rats

After revealing BDNF as one component in hMNC ^apo sec^, BDNF levels in plasma of rats treated with hMNC ^apo sec^ were measured with ELISA 24 hours after i.p. administration. Plasma BDNF-levels were higher in rats treated with hMNC ^apo sec^ compared to those treated with control medium (n=3 for each compound). Twenty four hours after treatment with hMNC ^apo sec^, BDNF plasma levels were 72±5ng/mL (mean±SEM) compared to undetectably low BDNF levels in controls ( Figure 8b).

Apoptotic MNC-secretomes in experimental stroke Mixed Model Analysis (SAS output)
The data were analyzed using linear mixed models for the neuroscore on treatment group and time-point with the factor animal included as a random effect. The MIXED procedure in SAS 9.3 was used to perform the calculations. The raw output contains information on the model specifications, the estimated error variance and random effects variance, the estimated regression coefficients, the covariance structure of the model coefficients and type III F-tests for the hypotheses of no effect of either fixed effect or their interactions. An interaction plot was drawn using the GLM procedure. This plot shows the individual observations and their sample mean values in each group and for each time-point. The group labels 0,1,2 and 3 in the raw output refer to the treatment group in setting 1, the control group in setting 1, the treatment group in setting 2 and the control group in setting 2, respectively.
Original Western blots to Figure 5 
Expression of proteins involved in cytoprotective pathways in human Astrocytes and Schwann Cells 
Astrocytes (page 1) or Schwann Cells (page 2) were stimulated with hMNCapo sec, control medium (served as control to treatment) or positive control (control to the measured protein). Original blots for all measured proteins are given in this raw data set (pages 1 and 2). For each blot, lanes (1), (2), and (3) correspond to the groups medium control [(1)=control to treatment], human apoptotic MNC-secretomes [(2)=treatment] and positive control [(3)=recombinant protein]. Bands in each blot are shown for phosphorylated CREB, total-CREB, phosphorylated Erk1/2, total-Erk 1/2, phosphorylated HSP27, total-HSP27, phosphorylated cJun, total-cJun, phosphorylated Akt, and total-Akt. The molecular weight (kDa) for each protein can be seen under each blot. Ponceau staining was used as loading control for each group (1), (2), and (3) and suggest equal loading.
Original Western blots to Figure 6 
Expression of Phosphorylated CREB in Astrocytes and Neurons after stimulation with the active compound hMNCapo sec or control medium: 
Cultured human Astrocytes and Neurons were incubated with hMNCapo sec or control (cell culture-) medium at indicated concentrations. Original blots can be seen here. Ponceau staining shows equal loading.

## Discussion

For over a decade, MSCs have been known to have beneficial effects on the outcome of several disease entities ( Assmus *et al.*, 2011; Chen *et al.*, 2001; Crigler *et al.*, 2006; Lagasse *et al.*, 2000). In our previous studies we were able to show that apoptotic MNC-secretomes share some of the regenerative characteristics of stem cells and, based on the data presented in this work, considerably more. We show here that apoptotic MNC-secretomes derived from both rats (rMNC ^apo sec^) and humans (hMNC ^apo sec^) caused a reduction of lesion volumes in rats subjected to MCAO. Neurological evaluations revealed an improvement in motor and sensory function, which was not observed in the control group. Furthermore, apoptotic MNC-secretomes derived from humans (hMNC ^apo sec^) (i) activate several mechanisms ultimately leading to the expression of protective proteins in cultured primary human glial cells, such as astrocytes, Schwann cells and human neurons, and (ii) induce notable sprouting of neurites in primary neuron cultures. Additionally, hMNC ^apo sec^ contain BDNF and lead to increased presence of BDNF in plasma of rats treated with hMNC ^apo sec^. Neurotrophic factors and proteins involved in protective pathways seem to be triggering the therapeutic factors found in our experiments.

Crigler and coworkers described the ability of MSCs to express neuro-regulatory molecules and to promote neuronal cell survival ( Crigler *et al.*, 2006). In the literature, MSCs are described to home to injured areas and regenerate damaged tissue by either causing cytoprotection, anti-inflammation or by inducing activation of endogenous stem cells ( Gnecchi *et al.*, 2012; Siegel *et al.*, 2012; Williams & Hare, 2011). Recently, it became commonly accepted that possible stem cell effects are derived from the paracrine factors secreted by MSCs ( Di Santo *et al.*, 2009; Jayaraman *et al.*, 2013). This theory of paracrine factors aiding in regenerative processes emerges as a possible explanation for the therapeutic potential of apoptotic MNC-secretomes shown previously in myocardial infarction and, given our new data, in ischemic stroke ( Ankersmit *et al.*, 2009; Lichtenauer *et al.*, 2011a; Lichtenauer *et al.*, 2011b). The mechanisms of action seem to be a matter of immunomodulation and cytoprotection ( Ankersmit *et al.*, 2009; Lichtenauer *et al.*, 2011a; Lichtenauer *et al.*, 2011b). Cultured glial cells incubated with apoptotic MNC-secretomes revealed an upregulation of several proteins involved in conveying cytoprotective signals, such as CREB, HSP27, Erk 1/2, and Akt. These results suggest that apoptotic MNC-secretomes affect different pathways within the ischemic cascade, and most prominently they appear to act via anti-apoptotic pathways. In concert with this it is tempting to speculate that the enhanced cell survival of glial cells is beneficial for neurons since glial cells are known to support and protect neurons ( Ferrer *et al.*, 2001). In rats subjected to MCAO the overexpression of HSP27 resulted in a 30% reduction of infarct sizes ( van der Weerd *et al.*, 2010). The extracellular signal-regulated kinases Erk 1/2, part of the MAPK-families (mitogen activated protein kinase), are thought to play a role in cell survival and proliferation ( Roux & Blenis, 2004). Furthermore, activation of the prosurvival kinase Akt reduces the proapoptotic signaling that is triggered by ischemia. It is suggested that Akt activation protects against ischemic brain injury by suppressing the proapoptotic JNK3 (c-Jun N-terminal kinase-3) pathway ( Zhang *et al.*, 2007). The activation of the transcription factor CREB induces BDNF, which plays an important role in neuronal protection ( Ferrer *et al.*, 2001). Studying the brain’s ischemic cascade, where repair processes are initiated through the expression of several of these aforementioned survival proteins, an upregulation of protective proteins and pathways by a targeted treatment is obviously a welcome effect ( Moskowitz, 2010).

Our data on CREB phosphorylation and neurite sprouting also suggest direct positive effects of apoptotic MNC-secretomes on neurons. We show here that apoptotic MNC-secretomes contain BDNF and enhance systemic, most likely indirect, BDNF secretion in rats after injection with human apoptotic MNC-secretomes. Neurotrophic factors may play a critical role in the treatment of cerebral ischemia ( Abe, 2000). It was discussed in the literature that BDNF is among the most important neurotrophic factors due to its capability to promote neurogenesis and angiogenesis, to prevent neuronal cell death, and to modulate local inflammatory processes ( Jiang *et al.*, 2011; Ploughman *et al.*, 2009; Schäbitz *et al.*, 2007). In light of these data it seems arguable that apoptotic MNC-secretomes provide, at least in part, indirect protection in this experimental stroke model via BDNF. When we integrate all these recent findings into our suggested mode of action of apoptotic MNC-secretomes in ischemic stroke, we can, step by step, characterize it as a biological, battling the ischemic cascade on several fronts. As mentioned before, the overall mechanism of action of our compound could be linked to the release of paracrine factors ( Korf-Klingebiel *et al.*, 2008).

We decided to run two experimental settings in order to investigate (i) whether syngeneic rMNC ^apo sec^ are able to attenuate ischemic lesion volumes and (ii) to further define whether xenogenic hMNC ^apo sec^, produced according to GMP criteria and virus-inactivated, are equally potent as rMNC ^apo sec^. This extended two-step experimental approach was chosen because hMNC ^apo sec^ are very close to the final product that is intended for later clinical use. hMNC ^apo sec^ can be produced effectively and securely using whole blood, similar to blood products such as packed red cells. Also, allogeneic apoptotic MNC-secretomes derived from multiple healthy donors could be pooled and lyophilized. In the future, the proposed ability of hMNC ^apo sec^ to reduce ischemic injury in the early phases of stroke may even imply its use in combination with established therapeutic concepts such as arterial recanalization. Considering that inflammatory processes are particularly aggressive in ischemia associated with reperfusion, the suppression of inflammation provided by hMNC ^apo sec^ would be a fitting addition to reperfusion therapy ( Stowe *et al.*, 2009). Experiments investigating the anti-inflammatory action of apoptotic MNC-secretomes in our setting of experimental MCAO are subject to future studies. We agree that rat and human apoptotic MNC-secretomes are hardly comparable, representing a limitation of this study. In this preclinical setting of stroke, however, we were able to find similar effects through both. The intention of this study was to investigate whether there is any beneficial effect of MNC-secretomes in this preclinical setting of stroke. To avoid possible loss of potency in the xenogenic setting (hMNC ^apo sec^ in rat MCAO), we decided to use treatment twice instead of only once in the allogeneic experiments (rMNC ^apo sec^ in rat MCAO). This approach further allowed us to reduce the required amount of animals according to the principle of the three R´s (replacement, reduction and refinement) ( Richard, 2001). This small study with all its agreeable limitations was done to see if the promising data obtained from previous studies might be translatable into experimental ischemic stroke. We realize that larger animal studies with a more thorough observation according to STAIR criteria are needed to further uncover effects of hMNC ^apo sec^ in experimental stroke and also to set a time window at which this treatment is feasible ( Fisher *et al.*, 2009).

## Conclusion

We suggest that apoptotic MNC-secretomes have multifaceted, direct and indirect, neuroprotective characteristics acting through different ways in the ischemic cascade (inflammation, apopotosis, ischemia). Rats treated with apoptotic MNC-secretomes in this experimental stroke study expressed smaller lesion volumes than control animals and showed improvement in neurological function over time. Based on our findings, we believe that apoptotic MNC-secretomes derived from human blood can aid in the development of new treatment strategies in ischemic stroke.

## Data availability

*figshare*: Apoptotic MNC-secretomes in experimental stroke. doi: 10.6084/m9.figshare.1051645 ( Altmann *et al.*, 2014).
