# Supplementary material for: Secretomes of apoptotic mononuclear cells ameliorate neurological damage in rats with focal ischemia
Source: F1000Res. 2014 Oct 28;3:131. Originally published 2014 Jun 19. [Version 2] doi: 10.12688/f1000research.4219.2 (PMC4215751; doi:10.12688/f1000research.4219.2)

| **Model Information** | |
| --- | --- |
| **Data Set** | Altmann et al |
| **Dependent Variable** | Neuro_ |
| **Covariance Structure** | Variance Components |
| **Subject Effect** | Name |
| **Estimation Method** | REML |
| **Residual Variance Method** | Profile |
| **Fixed Effects SE Method** | Model-Based |
| **Degrees of Freedom Method** | Containment |

| **Class Level Information** | | |
| --- | --- | --- |
| **Class** | **Levels** | **Values** |
| **Name** | 16 | 9 10 11 12 13 14 15 16 1 2 3 4 5 6 7 8 |
| **time** | 3 | 3 2 1 |
| **Typ** | 2 | 1 0 |

| **Dimensions** | |
| --- | --- |
| **Covariance Parameters** | 2 |
| **Columns in X** | 12 |
| **Columns in Z Per Subject** | 1 |
| **Subjects** | 16 |
| **Max Obs Per Subject** | 3 |

| **Number of Observations** | |
| --- | --- |
| **Number of Observations Read** | 48 |
| **Number of Observations Used** | 48 |
| **Number of Observations Not Used** | 0 |

| **Iteration History** | | | |
| --- | --- | --- | --- |
| **Iteration** | **Evaluations** | **-2 Res Log Like** | **Criterion** |
| **0** | 1 | 102.61775800 |  |
| **1** | 1 | 93.96440449 | 0.00000000 |

| Convergence criteria met. |
| --- |

| **Covariance Parameter Estimates** | | |
| --- | --- | --- |
| **Cov Parm** | **Subject** | **Estimate** |
| **Intercept** | Name | 0.2374 |
| **Residual** |  | 0.2634 |

| **Fit Statistics** | |
| --- | --- |
| **-2 Res Log Likelihood** | 94.0 |
| **AIC (smaller is better)** | 98.0 |
| **AICC (smaller is better)** | 98.3 |
| **BIC (smaller is better)** | 99.5 |

| **Solution for Fixed Effects** | | | | | | | |
| --- | --- | --- | --- | --- | --- | --- | --- |
| **Effect** | **time** | **Typ** | **Estimate** | **Standard Error** | **DF** | **t Value** | **Pr > \|t\|** |
| **Intercept** |  |  | 5.0000 | 0.2502 | 14 | 19.99 | <.0001 |
| **time** | 3 |  | -0.1875 | 0.2566 | 28 | -0.73 | 0.4710 |
| **time** | 2 |  | 2.01E-15 | 0.2566 | 28 | 0.00 | 1.0000 |
| **time** | 1 |  | 0 | . | . | . | . |
| **Typ** |  | 1 | -0.06250 | 0.3538 | 28 | -0.18 | 0.8611 |
| **Typ** |  | 0 | 0 | . | . | . | . |
| **time*Typ** | 3 | 1 | -1.5625 | 0.3629 | 28 | -4.31 | 0.0002 |
| **time*Typ** | 3 | 0 | 0 | . | . | . | . |
| **time*Typ** | 2 | 1 | -1.0625 | 0.3629 | 28 | -2.93 | 0.0067 |
| **time*Typ** | 2 | 0 | 0 | . | . | . | . |
| **time*Typ** | 1 | 1 | 0 | . | . | . | . |
| **time*Typ** | 1 | 0 | 0 | . | . | . | . |

| **Covariance Matrix for Fixed Effects** | | | | | | | | | | | | | | |
| --- | --- | --- | --- | --- | --- | --- | --- | --- | --- | --- | --- | --- | --- | --- |
| **Row** | **Effect** | **time** | **Typ** | **Col1** | **Col2** | **Col3** | **Col4** | **Col5** | **Col6** | **Col7** | **Col8** | **Col9** | **Col10** | **Col11** |
| **1** | Intercept |  |  | 0.06259 | -0.03292 | -0.03292 |  | -0.06259 |  | 0.03292 |  | 0.03292 |  |  |
| **2** | time | 3 |  | -0.03292 | 0.06585 | 0.03292 |  | 0.03292 |  | -0.06585 |  | -0.03292 |  |  |
| **3** | time | 2 |  | -0.03292 | 0.03292 | 0.06585 |  | 0.03292 |  | -0.03292 |  | -0.06585 |  |  |
| **4** | time | 1 |  |  |  |  |  |  |  |  |  |  |  |  |
| **5** | Typ |  | 1 | -0.06259 | 0.03292 | 0.03292 |  | 0.1252 |  | -0.06585 |  | -0.06585 |  |  |
| **6** | Typ |  | 0 |  |  |  |  |  |  |  |  |  |  |  |
| **7** | time*Typ | 3 | 1 | 0.03292 | -0.06585 | -0.03292 |  | -0.06585 |  | 0.1317 |  | 0.06585 |  |  |
| **8** | time*Typ | 3 | 0 |  |  |  |  |  |  |  |  |  |  |  |
| **9** | time*Typ | 2 | 1 | 0.03292 | -0.03292 | -0.06585 |  | -0.06585 |  | 0.06585 |  | 0.1317 |  |  |
| **10** | time*Typ | 2 | 0 |  |  |  |  |  |  |  |  |  |  |  |
| **11** | time*Typ | 1 | 1 |  |  |  |  |  |  |  |  |  |  |  |
| **12** | time*Typ | 1 | 0 |  |  |  |  |  |  |  |  |  |  |  |

| **Covariance Matrix for Fixed Effects** | |
| --- | --- |
| **Row** | **Col12** |
| **1** |  |
| **2** |  |
| **3** |  |
| **4** |  |
| **5** |  |
| **6** |  |
| **7** |  |
| **8** |  |
| **9** |  |
| **10** |  |
| **11** |  |
| **12** |  |

| **Type 3 Tests of Fixed Effects** | | | | |
| --- | --- | --- | --- | --- |
| **Effect** | **Num DF** | **Den DF** | **F Value** | **Pr > F** |
| **Time** | 2 | 28 | 14.30 | <.0001 |
| **Typ** | 1 | 28 | 10.81 | 0.0027 |
| **time*Typ** | 2 | 28 | 9.67 | 0.0006 |


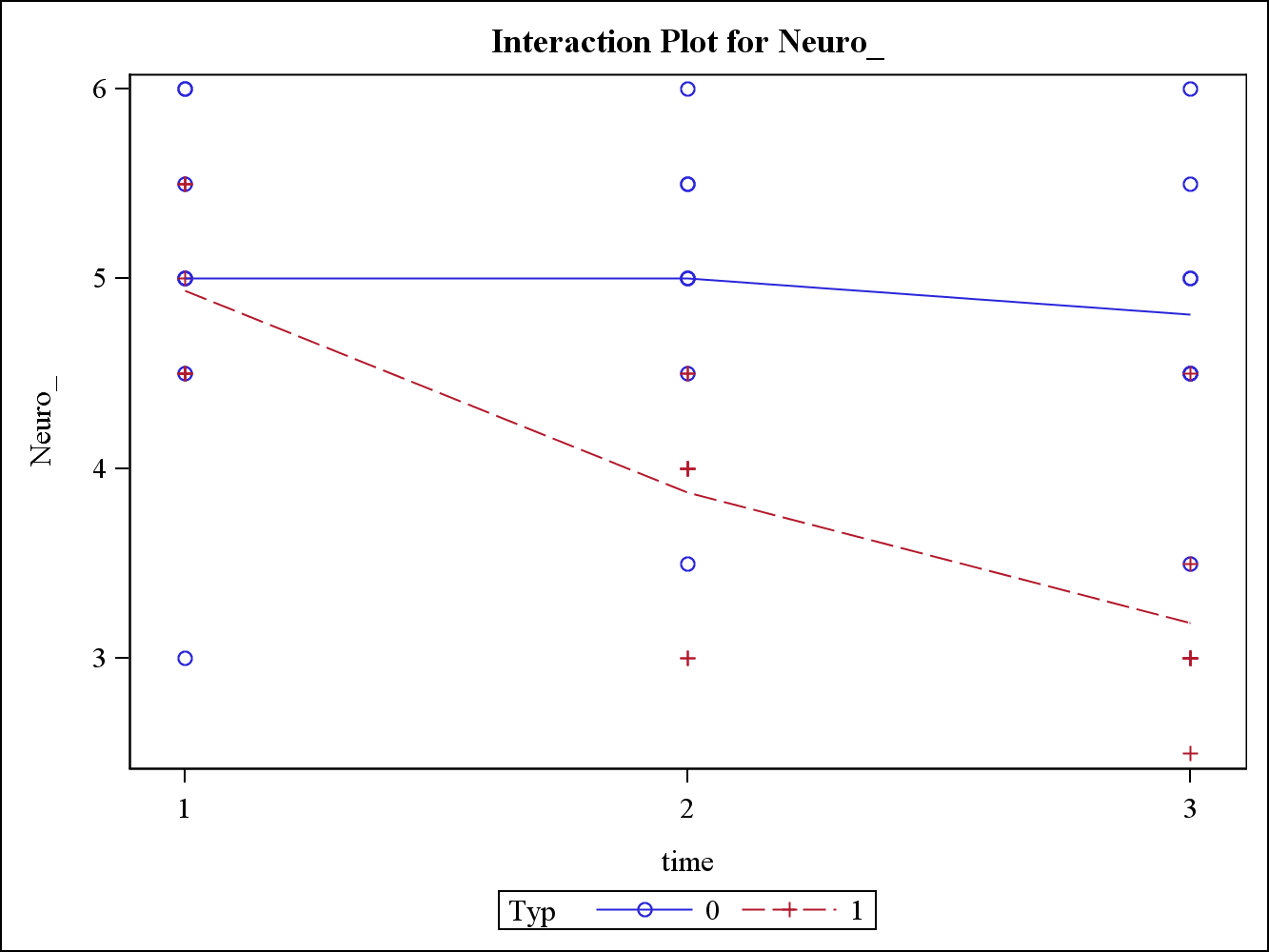


| **Model Information** | |
| --- | --- |
| **Data Set** | ALTMAN.DATA_LONG_SORT |
| **Dependent Variable** | Neuro_ |
| **Covariance Structure** | Variance Components |
| **Subject Effect** | Name |
| **Estimation Method** | REML |
| **Residual Variance Method** | Profile |
| **Fixed Effects SE Method** | Model-Based |
| **Degrees of Freedom Method** | Containment |

| **Class Level Information** | | |
| --- | --- | --- |
| **Class** | **Levels** | **Values** |
| **Name** | 14 | 24 25 26 27 28 29 30 17 18 19 20 21 22 23 |
| **time** | 3 | 3 2 1 |
| **Typ** | 2 | 3 2 |

| **Dimensions** | |
| --- | --- |
| **Covariance Parameters** | 2 |
| **Columns in X** | 12 |
| **Columns in Z Per Subject** | 1 |
| **Subjects** | 14 |
| **Max Obs Per Subject** | 3 |

| **Number of Observations** | |
| --- | --- |
| **Number of Observations Read** | 42 |
| **Number of Observations Used** | 42 |
| **Number of Observations Not Used** | 0 |

| **Iteration History** | | | |
| --- | --- | --- | --- |
| **Iteration** | **Evaluations** | **-2 Res Log Like** | **Criterion** |
| **0** | 1 | 91.23589930 |  |
| **1** | 1 | 89.60526381 | 0.00000000 |

| Convergence criteria met. |
| --- |

| **Covariance Parameter Estimates** | | |
| --- | --- | --- |
| **Cov Parm** | **Subject** | **Estimate** |
| **Intercept** | Name | 0.1190 |
| **Residual** |  | 0.4147 |

| **Fit Statistics** | |
| --- | --- |
| **-2 Res Log Likelihood** | 89.6 |
| **AIC (smaller is better)** | 93.6 |
| **AICC (smaller is better)** | 94.0 |
| **BIC (smaller is better)** | 94.9 |

| **Solution for Fixed Effects** | | | | | | | |
| --- | --- | --- | --- | --- | --- | --- | --- |
| **Effect** | **time** | **Typ** | **Estimate** | **Standard Error** | **DF** | **t Value** | **Pr > \|t\|** |
| **Intercept** |  |  | 4.5714 | 0.2761 | 12 | 16.56 | <.0001 |
| **time** | 3 |  | -0.5000 | 0.3442 | 24 | -1.45 | 0.1593 |
| **time** | 2 |  | 0.1429 | 0.3442 | 24 | 0.42 | 0.6818 |
| **time** | 1 |  | 0 | . | . | . | . |
| **Typ** |  | 3 | -714E-17 | 0.3905 | 24 | -0.00 | 1.0000 |
| **Typ** |  | 2 | 0 | . | . | . | . |
| **time*Typ** | 3 | 3 | -1.6429 | 0.4868 | 24 | -3.37 | 0.0025 |
| **time*Typ** | 3 | 2 | 0 | . | . | . | . |
| **time*Typ** | 2 | 3 | -1.4286 | 0.4868 | 24 | -2.93 | 0.0072 |
| **time*Typ** | 2 | 2 | 0 | . | . | . | . |
| **time*Typ** | 1 | 3 | 0 | . | . | . | . |
| **time*Typ** | 1 | 2 | 0 | . | . | . | . |

| **Covariance Matrix for Fixed Effects** | | | | | | | | | | | | | | |
| --- | --- | --- | --- | --- | --- | --- | --- | --- | --- | --- | --- | --- | --- | --- |
| **Row** | **Effect** | **time** | **Typ** | **Col1** | **Col2** | **Col3** | **Col4** | **Col5** | **Col6** | **Col7** | **Col8** | **Col9** | **Col10** | **Col11** |
| **1** | Intercept |  |  | 0.07625 | -0.05924 | -0.05924 |  | -0.07625 |  | 0.05924 |  | 0.05924 |  |  |
| **2** | time | 3 |  | -0.05924 | 0.1185 | 0.05924 |  | 0.05924 |  | -0.1185 |  | -0.05924 |  |  |
| **3** | time | 2 |  | -0.05924 | 0.05924 | 0.1185 |  | 0.05924 |  | -0.05924 |  | -0.1185 |  |  |
| **4** | time | 1 |  |  |  |  |  |  |  |  |  |  |  |  |
| **5** | Typ |  | 3 | -0.07625 | 0.05924 | 0.05924 |  | 0.1525 |  | -0.1185 |  | -0.1185 |  |  |
| **6** | Typ |  | 2 |  |  |  |  |  |  |  |  |  |  |  |
| **7** | time*Typ | 3 | 3 | 0.05924 | -0.1185 | -0.05924 |  | -0.1185 |  | 0.2370 |  | 0.1185 |  |  |
| **8** | time*Typ | 3 | 2 |  |  |  |  |  |  |  |  |  |  |  |
| **9** | time*Typ | 2 | 3 | 0.05924 | -0.05924 | -0.1185 |  | -0.1185 |  | 0.1185 |  | 0.2370 |  |  |
| **10** | time*Typ | 2 | 2 |  |  |  |  |  |  |  |  |  |  |  |
| **11** | time*Typ | 1 | 3 |  |  |  |  |  |  |  |  |  |  |  |
| **12** | time*Typ | 1 | 2 |  |  |  |  |  |  |  |  |  |  |  |

| **Covariance Matrix for Fixed Effects** | |
| --- | --- |
| **Row** | **Col12** |
| **1** |  |
| **2** |  |
| **3** |  |
| **4** |  |
| **5** |  |
| **6** |  |
| **7** |  |
| **8** |  |
| **9** |  |
| **10** |  |
| **11** |  |
| **12** |  |

| **Type 3 Tests of Fixed Effects** | | | | |
| --- | --- | --- | --- | --- |
| **Effect** | **Num DF** | **Den DF** | **F Value** | **Pr > F** |
| **Time** | 2 | 24 | 14.83 | <.0001 |
| **Typ** | 1 | 24 | 14.26 | 0.0009 |
| **time*Typ** | 2 | 24 | 6.73 | 0.0048 |


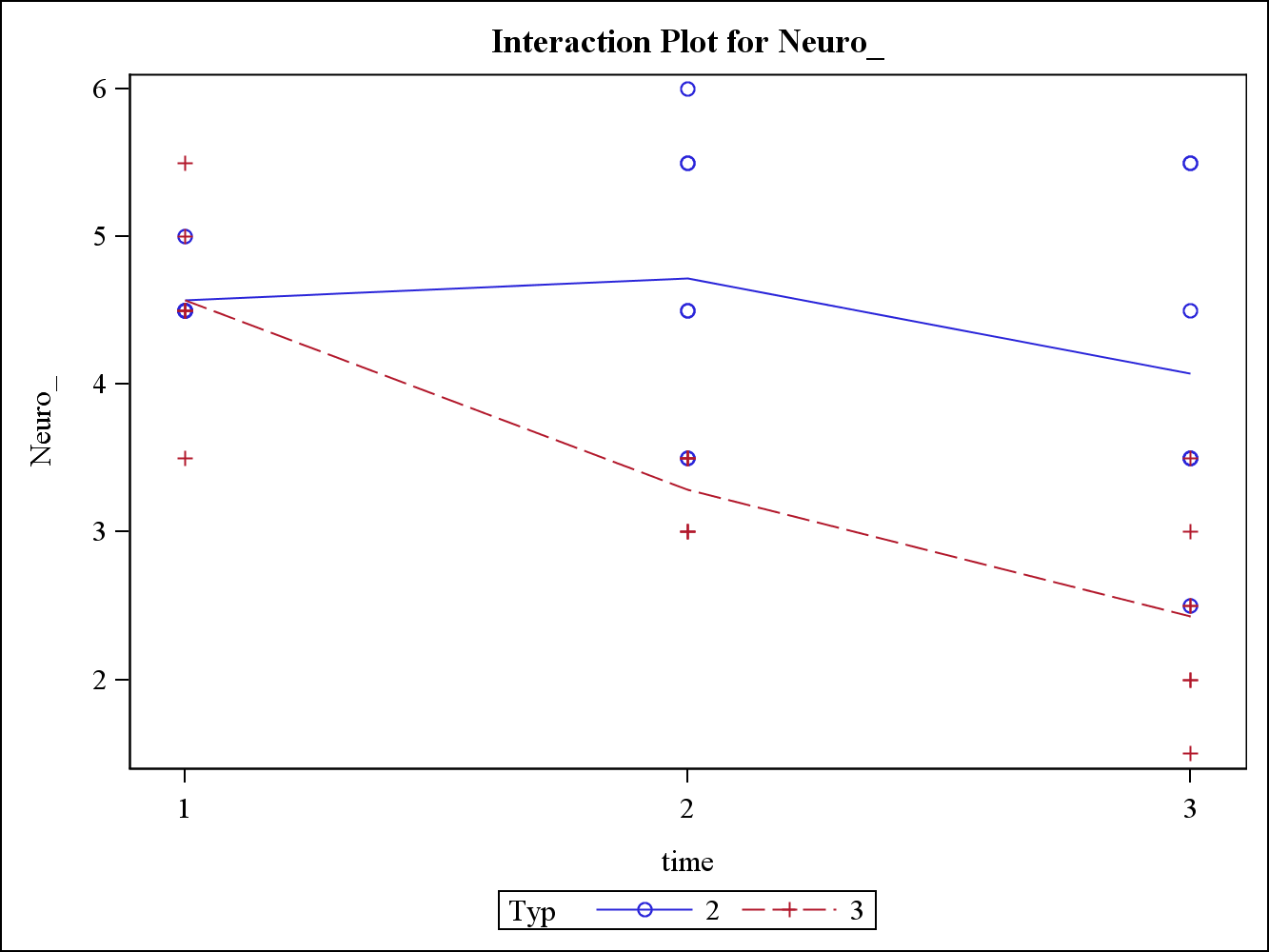

Supplement: Apoptotic MNC-secretomes in experimental stroke — Mixed Model Analysis (SAS output) The data were analyzed using linear mixed models for the neuroscore on treatment group and time-point with the factor animal included as a random effect. The MIXED procedure in SAS 9.3 was used to perform the calculations. The raw output contains information on the model specifications, the estimated error variance and random effects variance, the estimated regression coefficients, the covariance structure of the model coefficients and type III F-tests for the hypotheses of no effect of either fixed effect or their interactions. An interaction plot was drawn using the GLM procedure. This plot shows the individual observations and their sample mean values in each group and for each time-point. The group labels 0,1,2 and 3 in the raw output refer to the treatment group in setting 1, the control group in setting 1, the treatment group in setting 2 and the control group in setting 2, respectively. Original Western blots to Figure 5 Expression of proteins involved in cytoprotective pathways in human Astrocytes and Schwann Cells Astrocytes (page 1) or Schwann Cells (page 2) were stimulated with hMNCapo sec, control medium (served as control to treatment) or positive control (control to the measured protein). Original blots for all measured proteins are given in this raw data set (pages 1 and 2). For each blot, lanes (1), (2), and (3) correspond to the groups medium control [(1)=control to treatment], human apoptotic MNC-secretomes [(2)=treatment] and positive control [(3)=recombinant protein]. Bands in each blot are shown for phosphorylated CREB, total-CREB, phosphorylated Erk1/2, total-Erk 1/2, phosphorylated HSP27, total-HSP27, phosphorylated cJun, total-cJun, phosphorylated Akt, and total-Akt. The molecular weight (kDa) for each protein can be seen under each blot. Ponceau staining was used as loading control for each group (1), (2), and (3) and suggest equal loading. Original Western blots to Figure 6 Expression of Phosphorylated CREB [file f1000research-3-5935-s0000.tgz › Mixed_Model_SAS_output_Altmann_et_al.docx]
