# Supplementary material for: Secretomes of apoptotic mononuclear cells ameliorate neurological damage in rats with focal ischemia
Source: F1000Res. 2014 Oct 28;3:131. Originally published 2014 Jun 19. [Version 2] doi: 10.12688/f1000research.4219.2 (PMC4215751; doi:10.12688/f1000research.4219.2)

Original Western Blots (Figure 5)

Protective Proteins in Astrocytes

(1) Control medium

(2) $hMNC^{apo\ sec}$

(3) Positive control

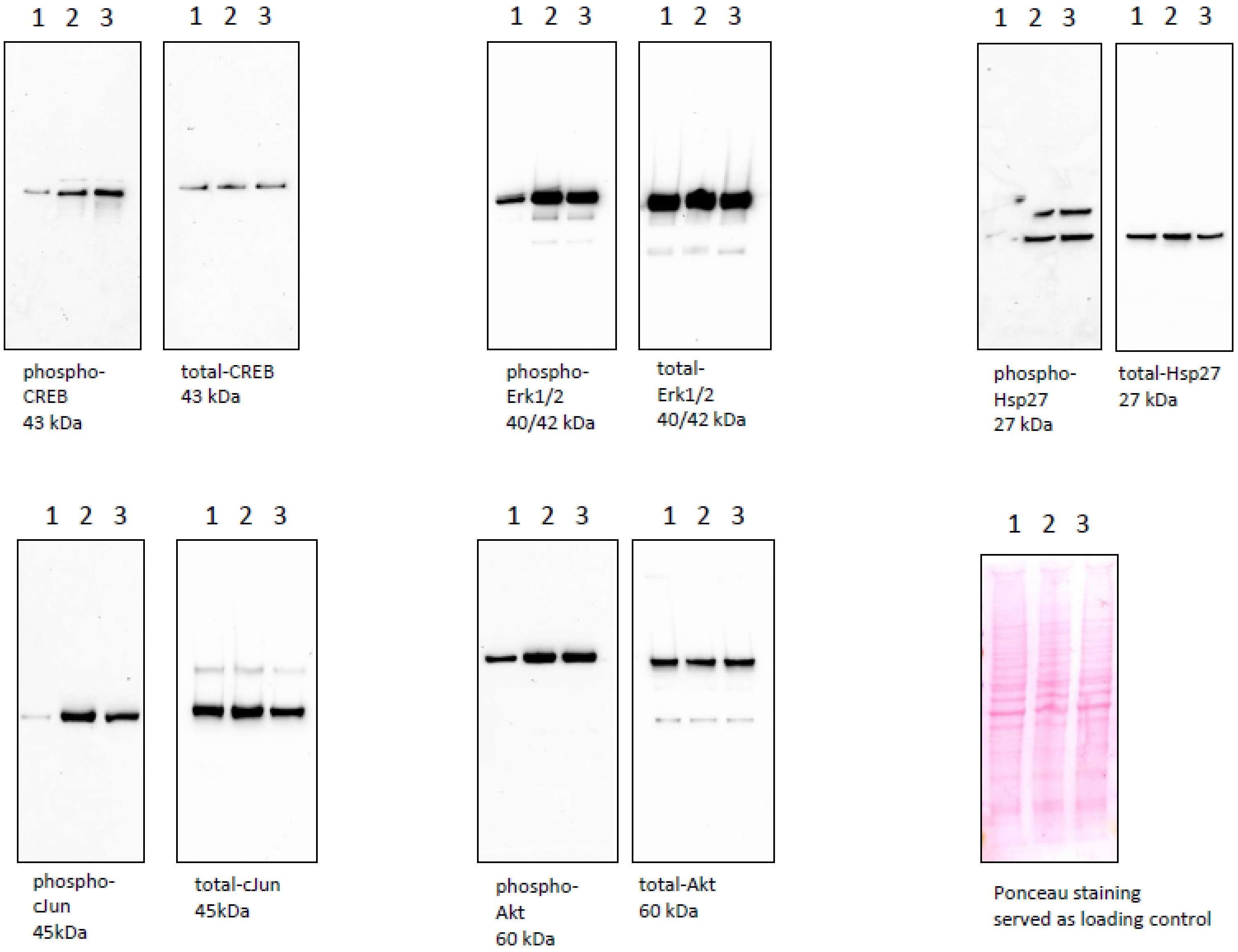

Original Western Blots (Figure 5)

Protective Proteins in Schwann Cells

(1) Control medium
(2) hMNC$^{apo\ sec}$
(3) Positive control

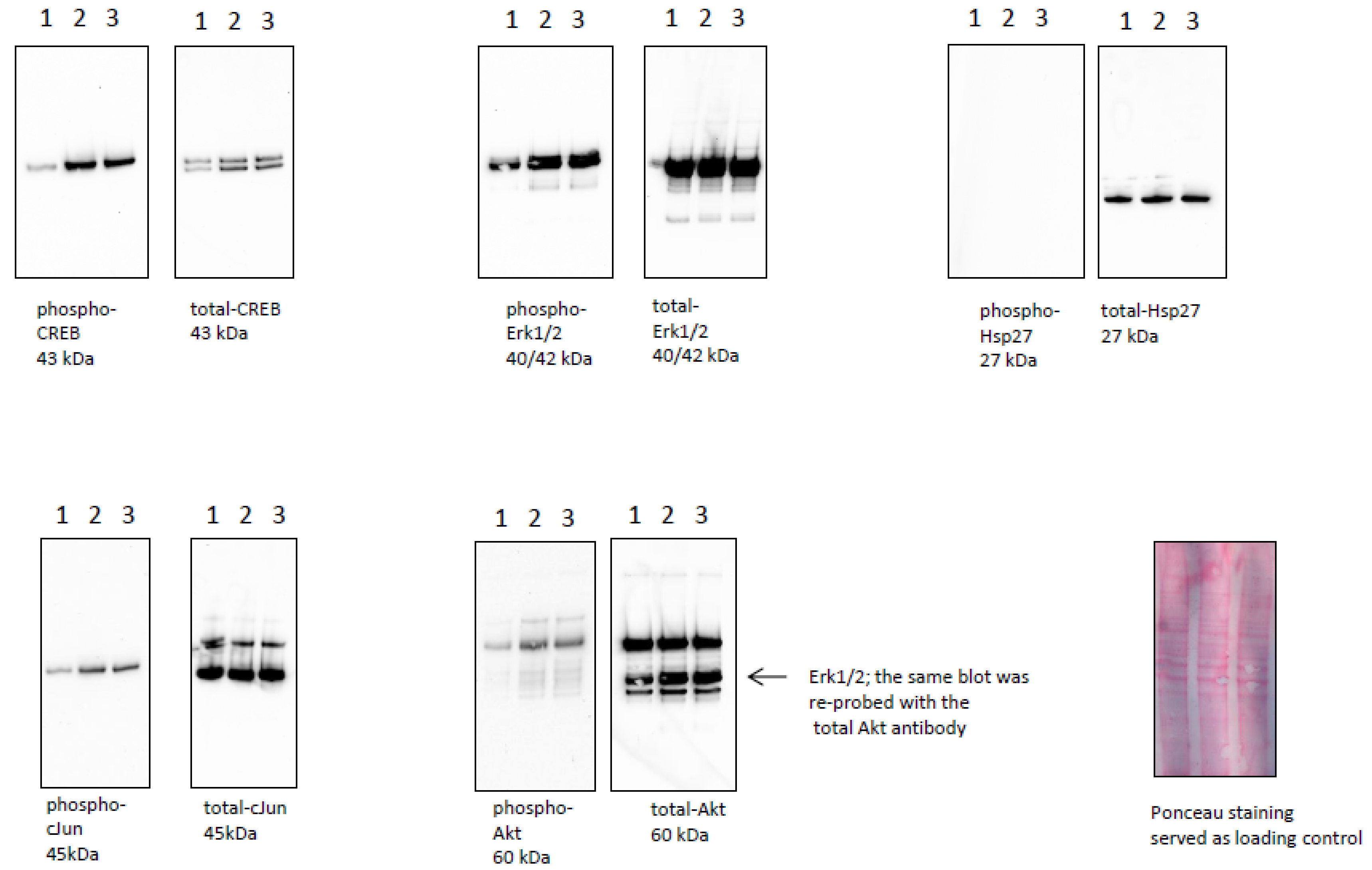

Original Western Blots (Figure 6)

phospho-CREB in Astrocytes and Neurons

Control Medium **$25x10^6$**
Control Medium **$2.5x10^6$**
Control Medium **$0.25x10^6$**
hMNC$^{apo\ sec}$ **$25x10^6$**
hMNC$^{apo\ sec}$ **$2.5x10^6$**
hMNC$^{apo\ sec}$ **$0.25x10^6$**

Astrocytes
phospho-CREB

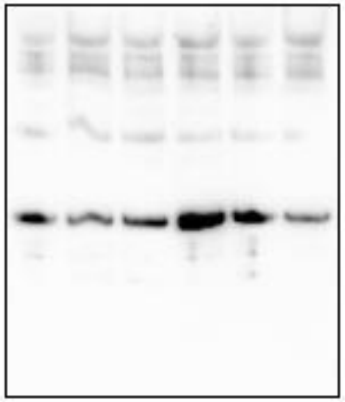

Ponceau
(loading control)

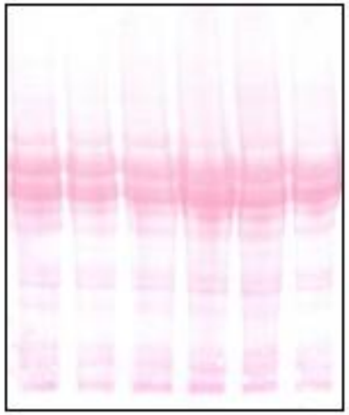

Control Medium **$25x10^6$**
Control Medium **$2.5x10^6$**
Control Medium **$0.25x10^6$**
hMNC$^{apo\ sec}$ **$25x10^6$**
hMNC$^{apo\ sec}$ **$2.5x10^6$**
hMNC$^{apo\ sec}$ **$0.25x10^6$**

Neurons
phospho-CREB

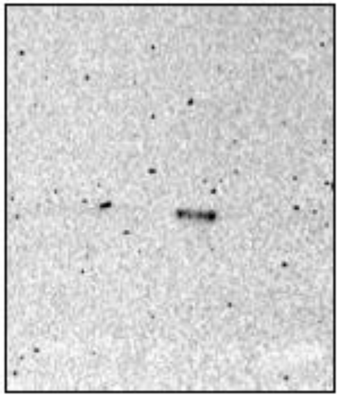

Ponceau
(loading control)

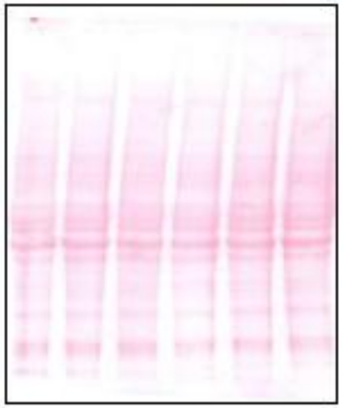

Supplement: Apoptotic MNC-secretomes in experimental stroke — Mixed Model Analysis (SAS output) The data were analyzed using linear mixed models for the neuroscore on treatment group and time-point with the factor animal included as a random effect. The MIXED procedure in SAS 9.3 was used to perform the calculations. The raw output contains information on the model specifications, the estimated error variance and random effects variance, the estimated regression coefficients, the covariance structure of the model coefficients and type III F-tests for the hypotheses of no effect of either fixed effect or their interactions. An interaction plot was drawn using the GLM procedure. This plot shows the individual observations and their sample mean values in each group and for each time-point. The group labels 0,1,2 and 3 in the raw output refer to the treatment group in setting 1, the control group in setting 1, the treatment group in setting 2 and the control group in setting 2, respectively. Original Western blots to Figure 5 Expression of proteins involved in cytoprotective pathways in human Astrocytes and Schwann Cells Astrocytes (page 1) or Schwann Cells (page 2) were stimulated with hMNCapo sec, control medium (served as control to treatment) or positive control (control to the measured protein). Original blots for all measured proteins are given in this raw data set (pages 1 and 2). For each blot, lanes (1), (2), and (3) correspond to the groups medium control [(1)=control to treatment], human apoptotic MNC-secretomes [(2)=treatment] and positive control [(3)=recombinant protein]. Bands in each blot are shown for phosphorylated CREB, total-CREB, phosphorylated Erk1/2, total-Erk 1/2, phosphorylated HSP27, total-HSP27, phosphorylated cJun, total-cJun, phosphorylated Akt, and total-Akt. The molecular weight (kDa) for each protein can be seen under each blot. Ponceau staining was used as loading control for each group (1), (2), and (3) and suggest equal loading. Original Western blots to Figure 6 Expression of Phosphorylated CREB [file f1000research-3-5935-s0000.tgz › Raw_Data_Figures_5_and_6_WB_Altmann_et_al_new.pdf]
