# Supplementary material for: Secretomes of apoptotic mononuclear cells ameliorate neurological damage in rats with focal ischemia
Source: F1000Res. 2014 Oct 28;3:131. Originally published 2014 Jun 19. [Version 2] doi: 10.12688/f1000research.4219.2 (PMC4215751; doi:10.12688/f1000research.4219.2)
Supplement: Peer review history

# Peer review: Referee response for version 2

**DOI:** 10.5256/f1000research.5935.r6551

**Published:** 2014-10-30

**Authors:** Michael Chopp, Hongqi Xin

**Affiliations:** 1 Department of Neurology, Henry Ford Hospital, Detroit, USA

The authors have addressed my concerns appropriately in the revised version.

We have read this submission. We believe that we have an appropriate level of expertise to confirm that it is of an acceptable scientific standard.

# Peer review: Referee response for version 1

**DOI:** 10.5256/f1000research.4517.r5562

**Published:** 2014-08-13

**Authors:** An Zhou

**Affiliations:** 1 Neuroscience Institute, Morehouse School of Medicine, Atlanta, GA, USA

In this work, the authors examined the therapeutic potential of secretory proteomes of apoptotic mononuclear cells (MNC ^apo sec^) in treating focal ischemic stroke, modeled on rats with permanent MCAO. They found that intraperitoneal administration of MNC ^apo sec^ prepared from either rat splenocytes or human circulating blood could significantly reduce MCAO-induce brain infarct. The authors also attempted to identify protein factors in the MNC ^apo sec^ that may play a role in the observed neuroprotection, and suggested BDNF. Further, in an *in vitro *setting, the authors analyzed changes in levels of a number of proteins known to mediate anti-apoptotic responses or neuronal regeneration in three different types of cell cultures, namely astrocytes, Schwann cells and neurons, upon treating the cultures with MNC ^apo sec^. An increase in some of selected proteins led to the suggestion of activation of several cytoprotective singnaling cascades.

The topic of the work is of significance in developing feasible therapeutics for stroke. The same group has previously published on the use of MNC ^apo sec^ in treating myocardial infarction. Thus the current findings are supportive of the potential of MNC ^apo sec^. Concerns exist, however, on several issues:

 

1. Lack of description of irradiation-treated MNC, especially in the splenocyte-derived cultures. Though referred to as apoptotic MNC, no experimental data were shown to demonstrate the induction and the extent of irradiation-induced apoptosis, for the period during which the conditioned media were collected. Do the two cell cultures (rat splenocytes-derived and human PBMC-derived) respond to irradiation with comparable characteristics? Are their secretomes comparable?
2. Please provide evidence on whether proteins in the intraperitoneally administered MNC ^apo sec^ may have reached the brain region of MCAO territory. This information would be instrumental in helping to understand the mechanisms that underlie the action of MNC ^apo sec^.
3. Post-MCAO neurological examinations were conducted at 6, 24 and 48 hours. The covered period was short in regard to the development of infarction and neurological impairments.
4. For the MNC ^apo sec^, please provide protein quantities administered. Different cell cultures or the same culture under different conditions, even with identical cell numbers, may differ in secretion rates.
5. The rationale for using three different cultures *in vitro *to investigate the cellular response upon MNC ^apo sec^ application is somewhat unconvincing. Astrocytes, Schwann cells and neurons may react to MNC ^apo sec^ administration differently in brain *in vivo*. Why not perform immunohistochemical analyses of proteins of interests on brain sections?
6. The determination of plasma BDNF levels included only 3 animals per data point. Was there a power determination to justify this small sample size?
7. Wording: in the title, the abstract and manuscript text, there appeared a somewhat interchangeable use of “ameliorate neurological damage”, “regenerative potential” and “promote neuronal cell survival”. Please revise to be more accurate and concise.

I have read this submission. I believe that I have an appropriate level of expertise to confirm that it is of an acceptable scientific standard, however I have significant reservations, as outlined above.

# Peer review: Referee response for version 1

**DOI:** 10.5256/f1000research.4517.r5568

**Published:** 2014-07-25

**Authors:** Michael Chopp, Hongqi Xin

**Affiliations:** 1 Department of Neurology, Henry Ford Hospital, Detroit, USA

The authors in this report examined the regenerative potential of conditioned medium derived from rat and human apoptotic mononuclear cells (MNC) in experimental stroke. They found that administration of rat as well as human apoptotic MNC-secretomes (rMNC ^apo sec^ and hMNC ^apo sec^) significantly reduced ischemic lesion volumes and improved neurological function after stroke in both treatment groups. Furthermore, co-incubation of human astrocytes, Schwann cells and neurons with hMNC-secretomes resulted in activation of several signaling cascades associated with the regulation of cytoprotective gene products and enhanced neuronal sprouting in vitro. These data indicate that apoptotic MNC-secretomes elicit neuroprotective effects on rats that have undergone ischemic stroke. Overall, this is an interesting study. However; there are some important pieces of information missing that should be addressed to improve the manuscript:

 

1. The authors should include the references for their protocol on inducing apoptotic rat MNCs, as well as the references for the protocol on rat MNC preparation. Data need be presented showing apoptosis and characterizing of their rat MNCs.
2. The authors mentioned in the Materials and Methods that they will discuss the rationale for the two-step-approach of their hMNC ^apo sec^ administration. However, this rationale is absent from the text. It is important to address why the authors employed two dosages instead of one dose (like the rMNC ^apo sec^).
3. In the part *“Neuronal sprouting assay”*, the authors should describe with more precision the methods they employed for measurement of neurite length.
4. In the part *“Determination of BDNF in rat plasma”*, the authors should include the dose and amount of hMNC ^apo sec ^they administered to rats.
5. In the Results “ *Apoptotic MNC-secretomes induce CREB phosphorylation and neuronal sprouting in human primary neurons and contain BDNF*”, the authors state that *“Interestingly, only high amounts of BDNF (356,6±13,66 pg/mL)were detected in hMNC ^apo sec^, suggesting an exclusive role for this neurotrophic factor in hMNC ^apo sec^ (Figure 7).”* But these data are not shown in Figure 7.
6. The authors used a comma symbol as decimal point, and the number of significant figures should be uniform throughout the manuscript.
7. Typos in the text, e.g. “rays” in third line of the conclusion part should be “ways”.

We have read this submission. We believe that we have an appropriate level of expertise to confirm that it is of an acceptable scientific standard, however we have significant reservations, as outlined above.

# Response

**Published:** 2014-10-15

**Authors:** Patrick Altmann

**Affiliations:** Medical University of Vienna, Austria

Thank you for your helpful report. Your suggestions have allowed us to make our manuscript better and present our data more accurately.

 

1. *The authors should include the references for their protocol on inducing apoptotic rat MNCs, as well as the references for the protocol on rat MNC preparation. Data need be presented showing apoptosis and characterizing of their rat MNCs.*
  Thank you for this suggestion. We conducted an additional experiment which allowed us to compare apoptosis rates of irradiated and non-irradiated MNCs using Annexin/PI staining and flow cytometry (please see “new” Figure 1). For our active compound, we found that 85% of rat MNCs that had been irradiated and cultured for 18 hours were apoptotic.
  Also, we referenced the piece of work where we generated and utilized rat apoptotic MNC‑secretomes for the first time.
2. *The authors mentioned in the Materials and Methods that they will discuss the rationale for the two-step-approach of their hMNC ^apo sec^ administration. However, this rationale is absent from the text. It is important to address why the authors employed two dosages instead of one dose (like the rMNC ^apo sec^).*
  Thank you for letting us know that this explanation has gotten lost somehow. Since we already had the most experience with apoptotic MNC-secretomes administered 40 minutes after myocardial infarction we decided to start our stroke research at this time window as well. This first investigation was mostly intended to show the potential of apoptotic MNC-secretomes in stroke rather than setting a particular time window for our treatment or being able to tell the best dosage for its application. We are aware of the limitations of this study design and we agree that additional experiments need to be done in order to see how the administration of apoptotic MNC-secretomes at different or, for that matter, later time points affect outcome after ischemic stroke.
  We applied the “human” dose twice in order to see if there was an additional benefit for the doubled dose. We agree, though, that rat and human apoptotic MNC-secretomes are hardly comparable, representing a limitation of this study. To avoid possible loss of potency in the xenogenic setting (human MNC-secretomes in rat MCAO) we decided to use treatment twice compared to only once in the allogeneic experiments (rat MNC-secretomes in rat MCAO), This approach further allowed us to reduce the required amount of animals according to the principle of the three R’s (replacement, reduction and refinement)[ref-1].
3. *In the part “Neuronal sprouting assay”, the authors should describe with more precision the methods they employed for measurement of neurite length.*
  Thank you for your comment. A blinded observer set a random area of the photographed cell culture (as seen in Figure 7b) using Adobe Photoshop software (i) of cells treated with human apoptotic MNC-secretomes and (ii) of cells treated with cell culture medium (control). The areas in each photograph measured 1420x2456 pixels. Subsequently, they picked and marked visible distinct, full-length and non-overlapping 30-35 neurites using ImageJ software. Another blinded investigator measured the lengths of these marked neurites using ImageJ software. We added this more detailed explanation to the Materials and Methods section.
4. *In the part “Determination of BDNF in rat plasma”, the authors should include the dose and amount of hMNC ^apo sec ^they administered to rats.*
  Thank you for making us aware of this. We added this to the manuscript. As in all studies here, we used secretomes of 12.5 million apoptotic MNCs.
5. *In the Results “Apoptotic MNC-secretomes induce CREB phosphorylation and neuronal sprouting in human primary neurons and contain BDNF”, the authors state that “Interestingly, only high amounts of BDNF (356,6±13,66 pg/mL)were detected in hMNC ^apo sec^, suggesting an exclusive role for this neurotrophic factor in hMNC ^apo sec^ (Figure 7).” But these data are not shown in Figure 7.*
  Thank you for showing this confusion. You can see that we now added a new panel to this Figure (now “new” Figure 8) with panel (a) showing how we only traced BDNF in our compounds and panel (b) showing BDNF levels of rats treated with our compounds.
6. *The authors used a comma symbol as decimal point, and the number of significant figures should be uniform throughout the manuscript.*
  Thank you for mentioning this. We replaced all commas by decimal points throughout the manuscript. Also, the number of significant figures are now uniform throughout the manuscript in order to increase readability.
7. *Typos in the text, e.g. “rays” in third line of the conclusion part should be “ways”.*
  Thank you. We proof-read the manuscript a couple more times **.**

[References]

[[1|type=journal|title=Animal research: the need for a middle ground.|authors=Smith/R|source=BMJ|year=2001|vol=322|issue=7281|fpage=248|lpage=249|pmid=11157509|pmcid=PMC1119509|doi=10.1136/bmj.322.7281.248]]

# Peer review: Referee response for version 1

**DOI:** 10.5256/f1000research.4517.r5480

**Published:** 2014-07-21

**Authors:** Johannes Boltze

**Affiliations:** 1 Department of Cell Therapies, Fraunhofer-Institut für Zelltherapie und Immunologie IZI, Leipzig, Germany

The article by Altmann and colleagues assesses the therapeutic impact of conditioned medium from both human and rodent apoptotic mononuclear cells (MNC). The paper reports highly interesting findings that not only contribute significantly to the understanding of adult cell-based therapies for ischemic diseases, but also shade some different light on the field.

Of particular value is the inclusion of a tailored statistical model by a professional statistician as this enhances reliability of results and their interpretation. Moreover, the method section is extensive, allowing easy reproduction of experiments by other labs. In summary, the article is clearly above the average quality of similar studies, highly informative, and relevant.

There are, however, a number of minor weak points that may be corrected or at least require some more detailed discussion.

1. Please report all available physiological parameters of animals during MCAO and details on the randomization strategy applied.
2. Why was the treatment started in a time window of <1h after stroke onset. This is clinically irrelevant and prevents collection of post-stroke baseline function. What was the rationale for applying secretomes from rat cells just one time, but two times from human cells? Why was the injection done i.p. and not i.v. or i.a. as common in the field?
3. Behavioral data should be presented as mean +/- standard deviation. For details, please see Carter (2013).
4. Please explain the rational of the surprisingly short post-stroke surveillance period of just 48 hours. Both STEPS and STAIR committees recommend at least 1 month of post-stroke observation in preclinical studies, so there should be strong argument for underscoring this significantly.
5. The discussion relies heavily on MSC which, potentially, is a bit distracting. There are ample papers discussing mechanisms of therapeutic impact by MNC, primarily from umbilical cord and bone marrow. The discussion should be directed towards those populations.
6. There are ample commas (as typical in German) in decimal numbers that should be replaced by periods.

I have read this submission. I believe that I have an appropriate level of expertise to confirm that it is of an acceptable scientific standard, however I have significant reservations, as outlined above.

# Response

**Published:** 2014-10-15

**Authors:** Patrick Altmann

**Affiliations:** Medical University of Vienna, Austria

Thank you for your valuable and kind comments. Having included them in our manuscript, we feel that we were able to further increase the value of our manuscript.

1. *Please report all available physiological parameters of animals during MCAO and details on the randomization strategy applied.*
  We agree that physiological parameters during surgery ensure the well-being of our animals, however after careful consideration we chose to avoid additional strain on our animals (through cannulizations of blood vessels, etc.). Preparing and designing our study, we found that other studies also abstained from invasive monitoring during surgery[ref-1] [ref-2] [ref-3]. To our knowledge, the model we used represents a well-established and often published procedure and therefore posesses predictable characteristics.
  Regarding the randomization process, the surgeon was blinded to treatment. After surgery, the animals‘ tails were labeled and observers blinded to treatment and previous scores assessed neurological function. We clarified this in the manuscript (Materials and Methods).
2. *Why was the treatment started in a time window of <1h after stroke onset. This is clinically irrelevant and prevents collection of post-stroke baseline function. What was the rationale for applying secretomes from rat cells just one time, but two times from human cells? Why was the injection done i.p. and not i.v. or i.a. as common in the field?*
  Thank you for mentioning this. We are aware of the time "issue“ and realize that our setting might be partially out of the ordinary. Since we already had the most experience with apoptotic MNC-secretomes administered 40 minutes after myocardial infarction we decided to start our stroke research at this time window as well. This first investigation was mostly intended to show the potential of apoptotic MNC-secretomes in stroke rather than setting a particular time window for our treatment or being able to tell the best dosage for its application. We are aware of the limitations of this study design and we agree that additional experiments need to be done in order to see how the administration of apoptotic MNC-secretomes at different or, for that matter, later time points affect outcome after ischemic stroke.
  We applied the “human” dose twice in order to see if there was an additional benefit for the doubled dose. We agree, though, that rat and human apoptotic MNC-secretomes are hardly comparable, representing a limitation of this study. To avoid possible loss of potency in the xenogenic setting (human MNC-secretomes in rat MCAO) we decided to use treatment twice compared to only once in the allogeneic experiments (rat MNC-secretomes in rat MCAO). This approach further allowed us to reduce the required amount of animals according to the principle of the three R’s (replacement, refuction and refinement) [ref-4].
  Concerning the injection route, we chose to administer our compounds intraperitoneally because (i) we felt it was easier to do, (ii) we had the most experience with applying medication via i.p. injections, and (iii) we considered it less invasive than intravenous application. Also, when we designed this study, we found some articles that did describe use of i.p. injections [ref-5] [ref-6] [ref-7].
3. *Behavioral data should be presented as mean +/- standard deviation. *
  Thank you for letting us know about this. We corrected this as requested.
4. *Please explain the rational of the surprisingly short post-stroke surveillance period of just 48 hours. Both STEPS and STAIR committees recommend at least 1 month of post-stroke observation in preclinical studies, so there should be strong argument for underscoring this significantly.*
  We absolutely agree that our small study does not meet STEPS/STAIR criteria. This very first investigation with all its agreeable limitations was done to see if the promising data obtained from previous studies in other fields of regenerative medicine might be translatable into experimental ischemic stroke. We realize that larger animal studies with a more thorough observation and surveillance in accordance with STAIR criteria are needed to further uncover effects of human apopototic MNC-secretomes in experimental stroke. We added this information to the Discussion (second paragraph).
5. *The discussion relies heavily on MSC which, potentially, is a bit distracting. There are ample papers discussing mechanisms of therapeutic impact by MNC, primarily from umbilical cord and bone marrow. The discussion should be directed towards those populations.*
  Thank you for showing this confusion. The first paragraph of the Discussion is intended to summarize what we know about MSCs and how we came to use secretomes of MNCs in our previous research and how we learned that they actually share some of the regenerative characteristics of MSCs. The second paragraph should point out how actually paracrine factors rather than stem cells themselves account for their regenerative properties and how this was observed in several studies. After that we discuss that our data indicates that neurotrophic factors involved in protective pathways seem to be triggering that therapeutic effect that we saw in our experiments. This is also mentioned in the Discussion and we included a new reference [ref-8].
6. *There are ample commas (as typical in German) in decimal numbers that should be replaced by periods.*
  Thank you. We replaced all ample commas in decimal numbers with periods.

[References]

[[1|type=journal|title=Treatment of cerebral ischemia by disrupting ischemia-induced interaction of nNOS with PSD-95. |authors=Zhou/L;Li/F;Xu/HB;Luo/CX;Wu/HY;Zhu/MM;Lu/W;Ji/X;Zhou/QG;Zhu/DY|source=Nat Med.|year=2010|vol=16|issue=12|fpage=1439|lpage=1443|pmid=21102461|doi=10.1038/nm.2245]]

[[2|type=journal|title=Panax notoginseng Attenuates the Infarct Volume in Rat Ischemic Brain and the Inflammatory Response of Microglia. |authors=Son/HY;Han/HS;Jung/HW;Park/YK|source=J Pharmacol Sci.|year=2009|vol=109|issue=3|fpage=368|lpage=379|pmid=19305121|doi=10.1254/jphs.08197FP]]

[[3|type=journal|title=Immunomodulatory effect of Hawthorn extract in an experimental stroke model. |authors=Elango/C;Devaraj/SN|source=J Neuroinflammation.|year=2010|vol=7|issue=97|pmid=21192826|pmcid=PMC3022819|doi=10.1186/1742-2094-7-97]]

[[4|type=journal|title=Animal research: the need for a middle ground.|authors=Smith/R|source=BMJ|year=2001|vol=322|issue=7281|fpage=248|lpage=249|pmid=11157509|pmcid=PMC1119509|doi=10.1136/bmj.322.7281.248]]

[[5|type=journal|title=Selective blockade of PGE2 EP1 receptor protects brain against experimental ischemia and excitotoxicity, and hippocampal slice cultures against oxygen-glucose deprivation. |authors=Ahmad/AS;Yun/YT;Ahmad/M;Maruyama/T;Doré/S|source=Neurotox Res.|year=2008|vol=14|issue=4|fpage=343|lpage=351|pmid=19073437]]

[[6|type=journal|title= Effects of citicoline used alone and in combination with mild hypothermia on apoptosis induced by focal cerebral ischemia in rats.|authors=Sahin/S;Alkan/T;Temel/SG;Tureyen/K;Tolunay/S;Korfali/E|source=J Clin Neurosci.|year=2010|vol=17|issue=2|fpage=227|lpage=231|pmid=20036128 |doi=10.1016/j.jocn.2009.05.016]]

[[7|type=journal|title=Candesartan and glycyrrhizin ameliorate ischemic brain damage through downregulation of the TLR signaling cascade.|authors=Barakat/W;Safwet/N;El-Maraghy/NN;Zakaria/MN|source=Eur J Pharmacol.|year=2014|vol=724|fpage=43|lpage=50|pmid=24378346 |doi=10.1016/j.ejphar.2013.12.032]]

[[8|type=journal|title=Bone marrow cells are a rich source of growth factors and cytokines: implications for cell therapy trials after myocardial infarction. |authors=Korf-Klingebiel/M;Kempf/T;Sauer/T;Brinkmann/E;Fischer/P;Meyer/GP;Ganser/A;Drexler/H;Wollert/KC|source=Eur Heart J.|year=2008|vol=29|issue=23|fpage=2851|lpage=2858|pmid=18953051|doi=10.1016/j.ejphar.2013.12.032|url=10.1093/eurheartj/ehn456]]
